# Computer Analysis of the Inhibition of ACE2 by Flavonoids and Identification of Their Potential Antiviral Pharmacophore Site

**DOI:** 10.3390/molecules28093766

**Published:** 2023-04-27

**Authors:** Andrey Bogoyavlenskiy, Madina Alexyuk, Pavel Alexyuk, Vladimir Berezin, Faisal A. Almalki, Taibi Ben Hadda, Alaa M. Alqahtani, Saleh A. Ahmed, Stefano Dall’Acqua, Joazaizulfazli Jamalis

**Affiliations:** 1 Research and Production Center for Microbiology and Virology, Almaty 050010, Kazakhstan; madina.a06@gmail.com (M.A.); pagenal@bk.ru (P.A.); vberezin359@gmail.com (V.B.); 2 Department of Pharmaceutical Chemistry, Faculty of Pharmacy, Umm Al-Qura University, Makkah 21955, Saudi Arabia; malkifaisal2@gmail.com (F.A.A.); amqahtani@uqu.edu.sa (A.M.A.); 3 Laboratory of Applied Chemistry & Environment, Faculty of Sciences, Mohammed Premier University, MB 524, Oujda 60000, Morocco; 4 Department of Chemistry, Faculty of Applied Science, Umm Al-Qura University, Makkah 21955, Saudi Arabia; saahmed@uqu.edu.sa; 5 Department of Pharmaceutical and Pharmacological Sciences, University of Padova, Via Marzolo 5, 35121 Padova, Italy; stefano.dallacqua@unipd.it; 6 Department of Chemistry, Faculty of Science, Universiti Teknologi Malaysia, UTM, Johor Bahru 81310, Johor, Malaysia

**Keywords:** plant compounds, flavonoids, inhibition of the ACE2 protein, docking, POM (Petra/Osiris/Molinspiration) analyses

## Abstract

In the present study, we investigated the antiviral activities of 17 flavonoids as natural products. These derivatives were evaluated for their in vitro antiviral activities against HIV and SARS-CoV-2. Their antiviral activity was evaluated for the first time based on POM (Petra/Osiris/Molispiration) theory and docking analysis. POM calculation was used to analyze the atomic charge and geometric characteristics. The side effects, drug similarities, and drug scores were also assumed for the stable structure of each compound. These results correlated with the experimental values. The bioinformatics POM analyses of the relative antiviral activities of these derivatives are reported for the first time.

## 1. Introduction

The renin-angiotensin-aldosterone system (RAAS) is one of the human body’s main systems and is of crucial importance in the regulation of most physiological and pathophysiological conditions, including vascular tone and blood pressure (BP), remodeling of the vascular wall and heart muscle, mechanisms of development and progression of atherosclerosis, glomerulosclerosis, and other pathologies [1,2,3,4,5]. Hyperactivity of the local (tissue) RAAS, the components of which are synthesized directly in various organs and tissues (heart, kidneys, eyes, adipose tissue, pancreas), can cause Kahn, Liddle, and Bartter syndromes, ischemic nephropathy, and diabetes mellitus [5,6]. This system was especially promising in 2020 after the discovery that one of the receptors of the SARS-CoV-2 virus is the angiotensin-converting enzyme 2 (ACE2), binding to which promotes the transport of the virus into the host cell [7,8]. Due to the widespread prevalence of COVID-19 around the world, the question of a detailed study of one of the key links in the pathogenesis of coronavirus infection, involving the ACE2 protein, is especially relevant. A detailed study of the enzyme, which is a receptor on the surface of various tissues and normally converts angiotensin II into angiotensin, has led to ambiguous conclusions. Being non-tissue-specific, the receptor is widely distributed in the heart, kidneys, small intestine, ovaries, thyroid gland, and adipose tissues. In addition to its direct bioregulatory function, it suppresses inflammation, mainly in lung tissue, participates in the transport of amino acids, and supports the vital activity of the intestinal microbiome. Thus, the ACE2 protein plays an essential role in several metabolic processes in the body. Simultaneously, along with performing many positive functions, the ACE2 protein promotes binding and transport to the host cell of the SARS-CoV-2 virus, which makes it possible to consider it a target for influencing coronavirus infection [9,10,11,12,13,14,15].

The use of inhibitors that affect the renin–angiotensin system may be a promising therapeutic strategy for combating coronavirus infection. Preliminary data on the use of ACE2 inhibitors, drugs containing this receptor in circulatory form, and angiotensin receptor II blockers indicate their effectiveness and the possibility of improving the condition and prognosis of patients with coronavirus infection taking ACE2 inhibitors [10,11].

Thus, when developing drugs to combat coronavirus infection, inhibitors of the ACE2 protein can be considered a promising group of compounds for the creation of new antiviral agents. The search for effective ACE2 blockers among various natural compounds can be conducted empirically using traditional viral models. However, this approach requires a large amount of work and considerable material costs. Therefore, in the first stage of assessing the potential capabilities of compounds, it seems appropriate to simulate their activity by molecular docking using computer programs. The promising structures selected based on the results of such a study can then be studied in more detail using appropriate experimental models, which will reduce both material costs and development time [16,17,18]. 

In the present work, computer docking and POM analyses of several polyphenolic and triterpenoid compounds were carried out and their possible effect on angiotensin converting enzyme 2 (ACE2), which contributes to the transport of the COVID-19 virus into the host cell, was evaluated. The POM theory was of great help in the identification of two antiviral pharmacophore sites (O ^δ−^, O′ ^δ−^, O″ ^δ−^) that are regenerated from parent flavonoids and their metabolites.

## 2. Results and Discussion

### 2.1. Docking Analysis

**Figure 1:**
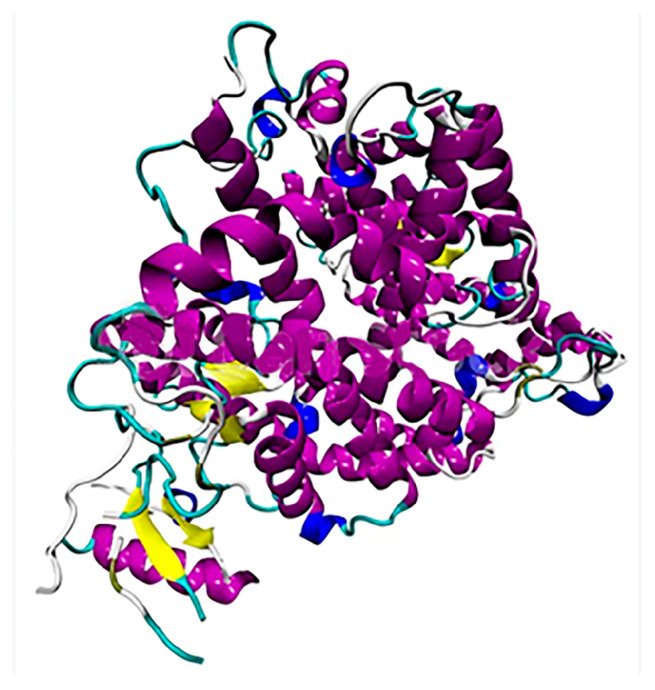
The structure of the ACE 2 protein.

The choice of the ACE2 protein model was associated with the possible determination of the mechanism of action of the ligand on either orthosteric or allosteric sites of ACE2 protein activity. Therefore, the Native Human Angiotensin Converting Enzyme-Related Carboxypeptidase (ACE2)-1R42 model was chosen to analyze possible inhibition (Figure 1) [19].

A computer simulation of the interaction of 50 plant compounds of various chemical natures with the ACE2 protein was carried out, and the binding activity of the studied structures with the protein was evaluated. The analysis considered the position at which the binding energy of the protein to the ligand was the best (lowest in kcal/mol).

The interaction activity of the analyzed drugs was compared with that of standard drugs used to inhibit ACE2. Computer analysis of the selected compounds revealed that the energy used for the ligand binding to the protein ranged from −7 to −10 kcal/mol (Table 1).

Based on the fact that the selected standard comparison drugs used to inhibit ACE2 in order to reduce pressure had binding energy ranging from −6 to −8 kcal/mol (captopril, MLN4760, Lisinopril, Amlodipin), we determined the cut-off value of the binding energy of the protein to the ligand as −7.4 kcal/mol. The use of this threshold value allowed us to identify 17 compounds with potentially high inhibitory properties in relation to the ACE2 protein (Table 1).

Compounds with a binding energy of the protein to the ligand below the selected cut-off value were divided into two equal groups, the first of which mainly interacted with the second allosteric activity center of the ACE2 protein and the second of which mainly interacted with the third allosteric activity center (Table 2 and Table S1).

The RAAS is of paramount importance in the regulation of most physiological and pathophysiological conditions, from the level of blood pressure (BP) to the mechanisms of development and progression of atherosclerosis [1,2,3,4]. Therefore, the main enzymes of this system, as a rule, can affect both orthostatic and allosteric regulation. This feature of angiotensin-converting enzyme type 2 (ACE2), which has an orthosteric and three allosteric sites of activity [20,21], creates both pros and cons in the search for new compounds affecting this protein using computer modeling methods.

Highly conserved amino acid residues Lys 31, Glu35, Asp38, Met82, and Lys353 were identified in the ACE 2 structure, four of which were located in the N-terminal part of the ectodomain of the enzyme responsible for interaction with the COVID-19 S protein [22]. Therefore, ligand binding to amino acids ACE2 30–41 a.r.; 82–84 a.r.; 353–357 a.r. will directly affect the possibility of S protein SARS-CoV-2 attachment to ACE2 during invasion of the host cell. At the same time, it was found that the spike protein from SARS-CoV-2 attaches to ACE 2 in a place other than the active site of the peptidase ACE2 [23], which somewhat reduces the possibility of using statins to alter the course of coronavirus infection.

The search for compounds affecting the orthosteric site of ACE2 showed that these are mainly short peptides or drugs, based on the use of monoclonal or polyclonal antibodies [20,21]. Simultaneously, the presence of three allosteric sites located on both sides of the orthosteric site and having an additional field for interaction with amino acids creates additional opportunities for identifying new compounds capable of suppressing the sorption of SARS-CoV-2 on the surface of competent cells. In our study, 17 compounds that can effectively interact with the ACE 2 molecule were identified. Analysis of the ability to form hydrogen bonds or bind via Van der Waals forces with enzyme molecules showed that the studied compounds were clearly divided into two main groups. The first includes a group of flavones that can interact with sites from one to several amino acids of the allosteric site of the ACE2 molecule. The second group of compounds not only interacts with amino acids of the allosteric site of the enzyme molecule but is also able to form bonds of various origins with amino acids of the orthosteric site of ACE2.

Thus, it was shown that computer modeling makes it possible not only to detect whether a ligand is able to interact with a particular protein but also to evaluate the mechanism of action of such compounds. The results obtained can be used for the preliminary assessment of the activity of compounds in the development of new effective drugs.

### 2.2. POM Analyses

POM Theory (Petra/Osiris/Molinspiration), which was proposed by a group led by Taibi Ben Hadda in collaboration with the American NCI and TAACF, has led to great success in the pharmacology and drug-design fields of antibacterial, antifungal, antiviral, and antiparasitic agents [24,25,26,27,28,29,30,31,32,33,34,35,36,37,38,39,40,41,42,43,44,45,46,47,48,49,50,51,52,53,54,55,56,57,58,59,60,61,62,63,64,65,66,67,68,69,70,71,72,73,74,75,76,77,78,79,80,81,82,83,84,85,86,87,88,89,90,91,92]. 

**Figure 2:**
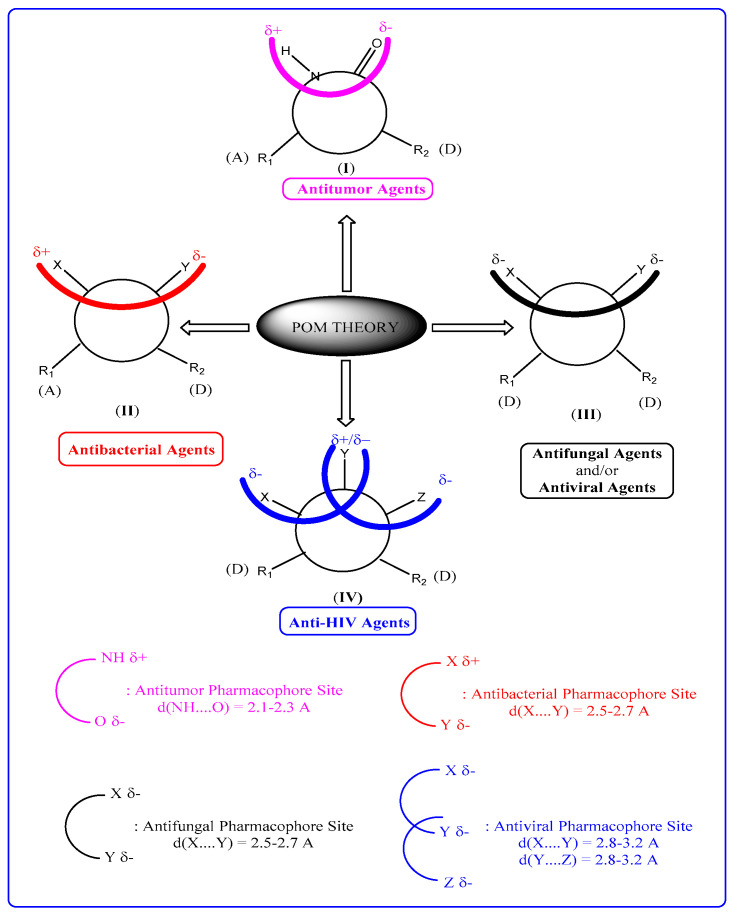
The Concept and Applications of POM Theory [78].

Here, we treated a series of natural products 1–17 with the goal of identifying their antiviral pharmacophore sites according to the POM organigram. The identification of pharmacophore sites for these compounds was derived from the physical and chemical properties of the tested compounds using a bioinformatics POM platform (Figure 2). 

#### 2.2.1. Osiris Calculations of Compounds 1–17

The results of the pharmacokinetic properties and bioactivity score analysis are shown in the supplementary information, Figure S1 and Table S2. The drug score was very encouraging (30% < DS < 75%). The toxicity risks and molecular characteristics of 1–17 were estimated. All structures examined were found to be non-tumorigenic and non-toxic-reproductive, but mutagenic. It seems that only 2 of the 17 compounds were safe, and the majority of compounds 1–17 exhibited side effects. This led us to find more indications of metabolites with better scores.

#### 2.2.2. Molinspiration Calculations of Compounds 1–17

The result of the Molinspiration analysis is presented in the supplementary information, Table S3. These bioactivity scores could be categorized as active (if the score was >0), moderately active (if the score was −5.0 to 0.0), or inactive (if the score was less than −5.0). Corroboration of all bioactivity parameters showed that the majority of the flavonoid derivatives were biologically active against all of the enzymes involved (Table S4).

#### 2.2.3. Atomic Charge Calculation of Compounds 1–17

**Figure 3:**
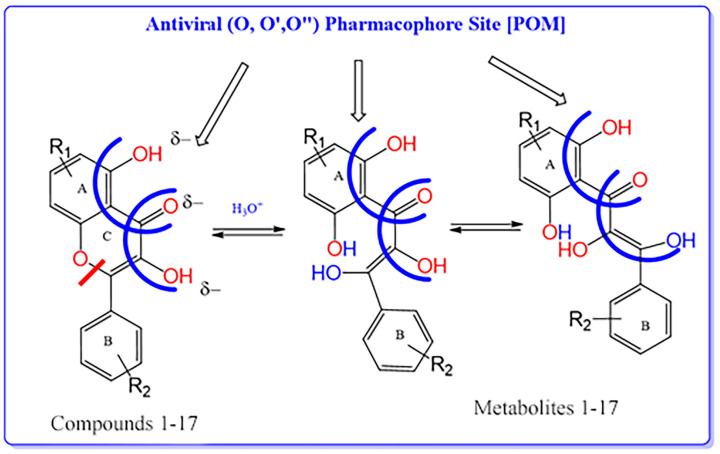
Proposed mechanism of opening and closing central ring C and identification of the antiviral pharmacophore sites for compounds 1–17 [POM].

According to the results of the calculations of atomic charge shown in the supplementary information, Figure S2, it is clear that most compounds 1–17 have an (O ^δ−^, O′ ^δ−^, O″ ^δ−^) antiviral pharmacophore site. Because of this, most hits are related to antiviral agents rather than antifungal or antibacterial agents. As a result, it was identified as a pharmacophore site with antiviral properties for compounds 1–17 (Figure 3).

#### 2.2.4. Identification of Antiviral Pharmacophore Sites

The flavonoids 1–17 have been described as subjects of opening/closing ring C and present moderate to good antiviral activity scores, according to the results of the experimental and theoretical POM calculations (Figure 3).

## 3. Materials and Methods

Molecular modeling of the interaction of the analyzed compounds with the ACE 2 protein was carried out using a computer analysis of the ligand-binding activity to the target. Such modeling makes it possible to place the ligand in the preferred binding site of the protein and to evaluate the specificity and binding efficiency by determining the interaction energy of the components. The information obtained through molecular docking can be used to predict the free binding energy and stability of the complexes. The main goal of molecular docking is to obtain a protein–ligand complex with an optimized conformation and lower binding free energy. 

For the practical application of molecular docking, a data bank is required to search for a target with the appropriate PDB format and a methodology for preparing the ligand in the form of a PDB file. Various programs are available in which the ligand can be converted to the PDB format. In our case, ligand preparation was carried out in the PyMol program 2.5.2 (https://pymol.org/2/, accessed 2 February 2022) [93,94,95,96,97,98,99]. Before direct modeling, the target and the ligand were prepared. To do this, the established structure of the selected protein underwent significant changes and was converted to the PDBQT format using the AutoDock Tools program 1.5.6. Subsequently, each ligand was also converted to the PDBQT format. The computer simulation itself was carried out using the AutoDock Vina program 1.1.2 (http://vina.scripps.edu/, accessed 2 February 2022), as well as external tools such as AutoDock Tools (http://mgltools.scripps.edu/downloads, accessed 2 February 2022) [98,99]. 

In this program, the protein combines with one of the ligands to form a protein–ligand complex. After entering the data, the program automatically generates a grid of the active center of the protein after which an algorithm is prescribed in the command line to determine the active sites of the ligand. As a result, the AutoDock Vina program 1,1,2 identified nine active ligand positions, with the best position being in the region with the lowest binding energy (BE) in kcal/mol. Visualization of the protein–ligand complex itself can be performed using various programs. We performed 2D visualization of the ACE2 protein with a ligand in the LigPlot+ program 2.2.5. This program allows for better visualization of the connection between the protein and ligand. This determines the amino acids that are directly linked to the ligand (https://www.ebi.ac.uk/thornton-srv/software/LigPlus/download2.html, accessed 2 February 2022) [98,99].

For a comparative study, 54 compounds were used, including four compounds used as blockers of the ACE2 protein at elevated pressure. The choice of compounds was determined based on previous studies (data are not presented) showing high efficacy of glycoside derivatives of flavonoids, methoxtflavonoids, and triterpene compounds.

## 4. Conclusions

We conducted a computational docking and POM study to identify the pharmacophore sites of 17 flavonoids, known as anti-viral agents. All flavonoids were successfully analyzed in silico for antiviral activity prediction, POM calculation, molecular docking, and pharmacokinetic properties. The insertion of various aliphatic and aromatic groups into the basic flavonoid structure can considerably improve its biological and antiviral activities. Antiviral prediction indicated that aliphatic/aromatic (1–17) derivatives exhibit potential antiviral effects. These findings were rationalized through molecular docking, which revealed the excellent antiviral efficacy of flavonoid derivatives. Many derivatives have shown outstanding binding energy and binding interactions with biotargets. Most flavonoid derivatives exhibited good potential in silico to inhibit HIV. The derivatives were unraveled in a stable binding conformation in the docked pocket, engaged by both hydrophobic and hydrophilic interactions. Future in vitro and in vivo studies are needed to determine whether these derivatives can be used to treat SARS-CoV-2 and HIV. The POM study confirmed the predominant antiviral profile of most compounds in series 1–17. This is highly encouraging to screen the antiviral hits as potential antiviral candidates.

## Figures and Tables

**Table 1:** Binding energy of the selected ligands with the studied protein.

No.	Ligand	Binding Energy (kcal/mol)
1	Rhamnetin (3,3′,4′,5-Tetrahydroxy-7-methoxyflavone)	−8.1
2	Patuletin (3,3′,4′,5,7-Pentahydroxy-6-methoxyflavone)	−7.7
3	Isorhamnetin (3,4′,5,7-Tetrahydroxy-3′-methoxyflavone)	−7.7
4	Tamarixetin (3,3′,5,7-Tetrahydroxy-4′-methoxyflavone)	−7.8
5	Quercetin (3,3′,4′,5,7-Pentahydroxyflavone)	−7.6
6	Corniculatusin (3,3′,4′,5,7-Pentahydroxy-8-methoxyflavone)	−8.2
7	Dillenetin (3,4-Dimethoxy-3,5,7-trihydroxyflavone)	−7.5
8	Nobiletin (3′,4′,5,6,7,8-Hexamethoxyflavone)	−7.8
9	Hesperidin (7-Rhamnoglucoside)	−9.6
10	Baicalein (5,6,7-Trihydroxyflavone)	−8.1
11	Ayanin (5,3′-Dihydroxy-3,7,4′-trimethoxyflavone)	−7.4
12	Azaleatin (3,3′,4′,7-Tetrahydroxy-5-methoxyflavone)	−7.6
13	Ombuin (4′,7-Dimethoxy-3,3′,5-trihydroxyflavone)	−7.8
14	Pachypodol (4′,5-Dihydroxy-3,3′,7-trimethoxyflavone)	−7.2
15	Retusin (5-Hydroxy-3,3′,4′,7-tetramethoxyflavone)	−7.3
16	Rhamnazin (Dimethoxyflavone)	−7.8
17	Eupatolitin (3,3′,4′,5-Tetrahydroxy-6,7-dimethoxyflavone)	−7.8
18	Captopril	−6.5
19	MLN4760	−7.4
20	Lisinopril	−8.0
21	Amlodipin	−7.3

**Table 2:** Features of the interaction of ACE2 protein with the ligand.

No.	Hydrogen Bonds	Van der Waals Force	Allosteric Site Surrounding Amino Acids Residues
1	Asp206, Trp566, Asn210	Asn397, Lys562, Ala99, Ala396, Glu208, Pro565, Leu95, Val212, Val209	2
2	Tyr196, Glu564, Ala396, Lys562, Glu208	Leu95, Pro565, Asp206, Glu98, Gly205, Gln98, Gln102, Tyr202	2
3	Gly208, Tyr196, Gly205, Lys502	Asp206, Ala99, Leu392, Leu95	2
4	Tyr385, Asp350, Asp382, Aka348	His378, Arg393, Phe390, Phe40, Asn394, His401	3
5	Asp206, Asn210, Trp566	Asn397, Lys562, Ala396, Gln208, Val212, Leu95, Val209, Pro565	2
6	Asp206, Asp210, Trp566	Ala396, Asn397, Lys562, Gln98, Pro565, Val209, Gln208, Leu95, Val212	2
7	Trp566, Lys562	Val209, Gln564, Pro565, Leu95, Gln98, Asp206, gln208, Gly205, Tyr196, Tyr202, Gln102	2
8	Asn210	Val212, Val209, Leu95, Pro565, Lys562, Gln205, Asp206, Tyr202, Tyr196, Gln208, Gln91, Lys94, Gln102	2
9	Ala248, Gln402, Glu375, Tyr385, Asp350, Ser47, Ser43	Trp69, Gly68, Phe40, Asp382, Asn394, Trp349, His401, His378, Thr347	3
10	Trp566, Ala396, Asp206	Lys562, Leu95, Val209, Asn210, Pro565, Glu564, Asn397	2
11	Tyr196	Trp203, Gln102, Tyr202, Gly205, Asp206, Gln98, Leu95, Lys562, Glu208	2
12	Asp509, Ser511, Tyr199, Tyr196, Gln102	Tyr510, Trp203, Asp514, Asp206, Tyr202	2
13	Asp350, Asp382, Ala348	Arg393, Phe390, Phe40, Asn394, His401, His378, Tyr385	3
14	Lys562, Tyr196	Trp566, Pro565, Val209, Gln208, Leu392, Leu95, Ala99, Glu208	2
15	Tyr196	Tyr202, Trp203, Asp206, Gly205, Leu95, Glu564, Lys562, Gln208, Gln98, Ala396	2
16	Trp566, Ala396, Gln102	Asn210, Val209, Asp206, Lys562, Pro565	2
17	Trp566, Glu564, Lys562, Gln98, Tyr196	Pro565, Leu95, Asp206, Gln102, Tyr202	2

## Data Availability

No new compounds reported.

## Supplementary Materials

The following supporting information can be downloaded at: https://www.mdpi.com/article/10.3390/molecules28093766/s1, Table S1a: 2D and 3D visualization of ligand binding to the protein under study; Table S1b: 2D and 3D visualization of ligand binding to the protein under study; Table S1c: 2D and 3D visualization of ligand binding to the protein under study; Table S2: Comparison of Toxicity Risks and Drug Score of compounds 1–17; Table S3: Molinspiration calculations for compounds 1–17; Table S4: Comparison of Molinspiration data for compounds 1–17; Figure S1: Osiris calculations of likeness and drug-score of compounds (1–17); Figure S2: Atomic charge calculations for compounds 1–17.
