# Supplementary material for: Computer Analysis of the Inhibition of ACE2 by Flavonoids and Identification of Their Potential Antiviral Pharmacophore Site
Source: Molecules. 2023 Apr 27;28(9):3766. doi: 10.3390/molecules28093766 (PMC10179817; doi:10.3390/molecules28093766)
Supplement: Supplementary file 1 [file molecules-28-03766-s001.zip › molecules-2368996-supplementary.pdf]

Computer Analysis Inhibition ACE2 by Flavonoids and Identification of their Potential Antiviral Pharmacophore Site

Andrey Bogoyavlenskiy [1,*], Madina Alexyuk [1], Pavel Alexyuk [1], Vladimir Berezin [1], Faisal A. Almalki [2], Taibi Ben Hadda [2,3,*], Alaa M. Alqahtani [2], Saleh A. Ahmed [4,5], Stefano Dall'Acqua [6], Joazaizulfazli Jamalis [7,*]

1 Research and Production Center for Microbiology and Virology, Almaty 050010, Kazakhstan; madina.a06@gmail.com (M.A.); pagenal@bk.ru (P.A.); vberezin359@gmail.com (V.B.)

2 Department of Pharmaceutical Chemistry, Faculty of Pharmacy, Umm Al-Qura University, Makkah 21955, Saudi Arabia; malkifaisal2@gmail.com (F.A.A.); amqahtani@uqu.edu.sa (A.M.A.)

3 Laboratory of Applied Chemistry & Environment, Faculty of Sciences, Mohammed Premier University, MB 524, Oujda 60000, Morocco

4 Department of Chemistry, Faculty of Applied Science, Umm Al-Qura University, Makkah 21955, Saudi Arabia; saahmed@uqu.edu.sa

5 Department of Pharmaceutical and Pharmacological Sciences, University of Padova, Via Marzolo 5, 35121 Padova, Italy; stefano.dallacqua@unipd.it

6 Department of Chemistry, Faculty of Science, Universiti Teknologi Malaysia, UTM, Johor Bahru 81310, Johor, Malaysia

* Correspondence: anpav_63@mail.ru (A.B.); taibi.ben.hadda@gmail.com (T.B.H.); joazaizulfazli@utm.my (J.J.)

Table S1a 2D and 3D visualization of ligand binding to the protein under study.

№	Compound	Visualization ACE2 + compound in PyMol
1	Rhamnetin	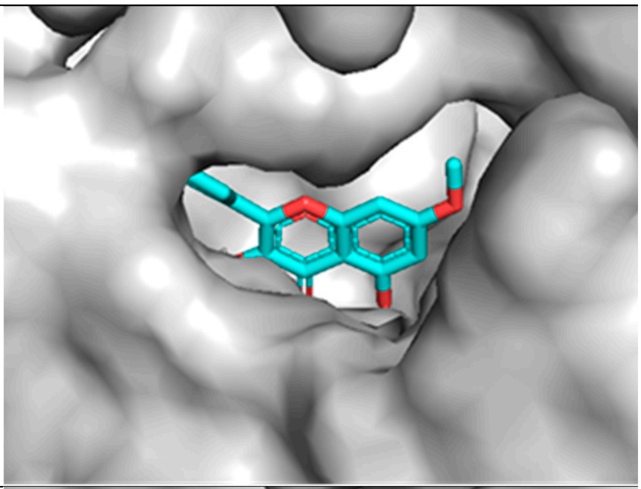
2	Patuletin	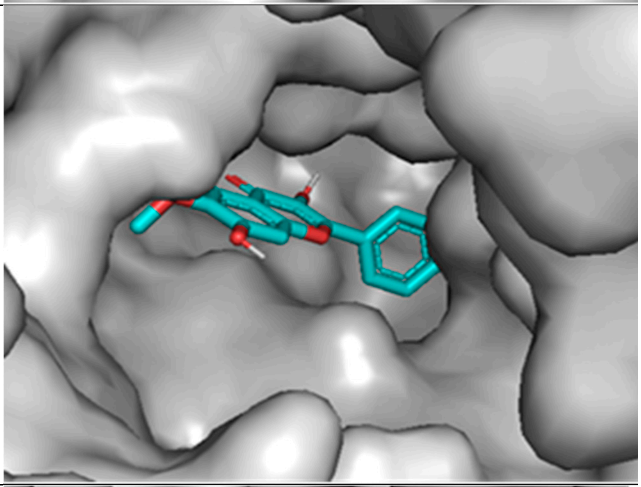
3	Isorhamnetin	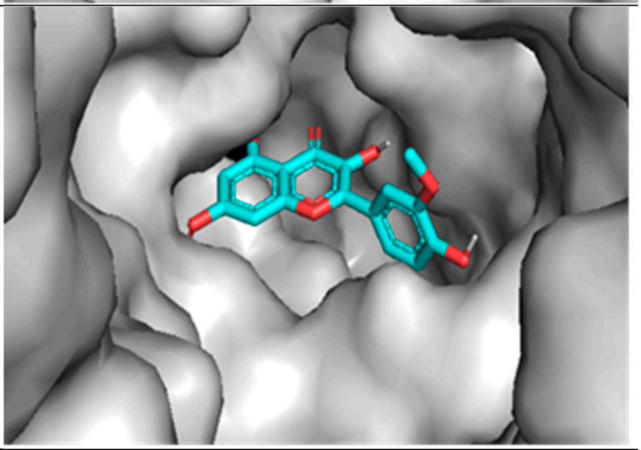

4	Tamarixetin	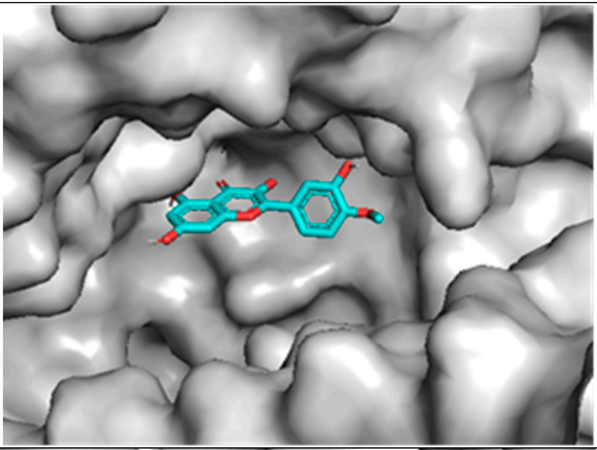
5	Quercetin	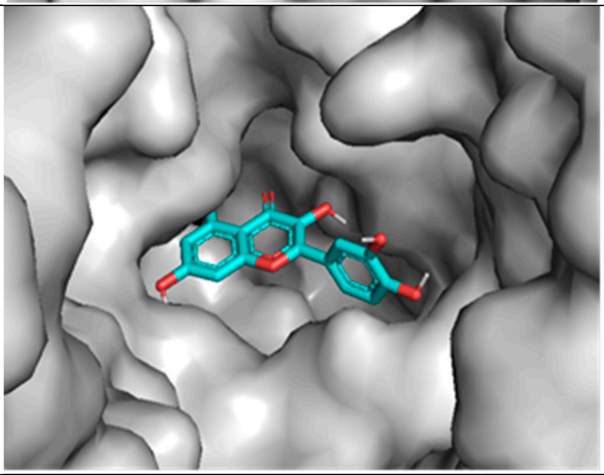
6	Corniculatus in	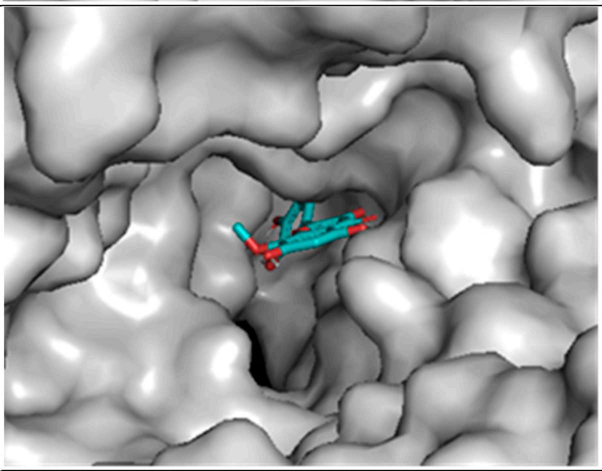
7	Dillenetin	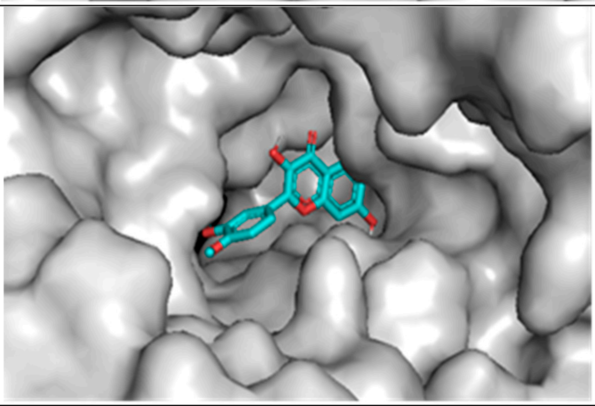

8	Nobiletin	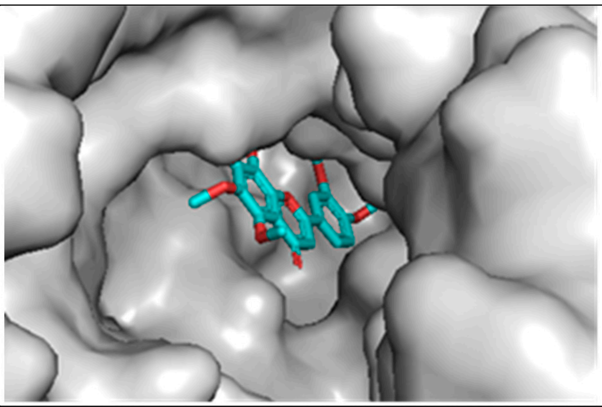
9	Hesperidin	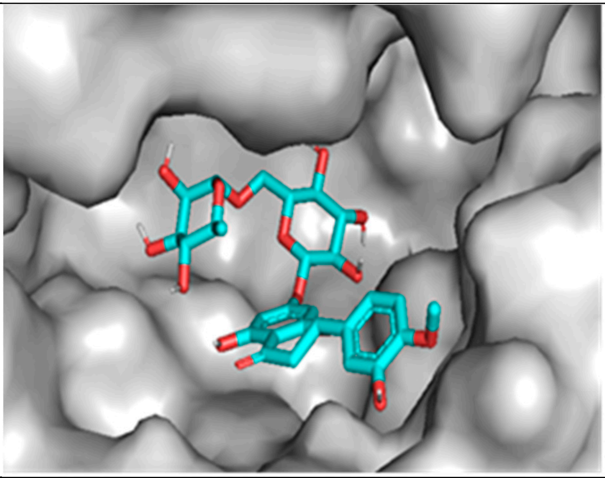
10	Baicalein	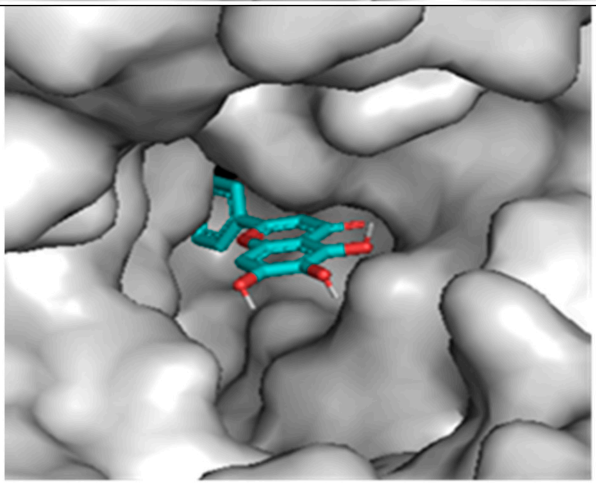
11	Ayanin	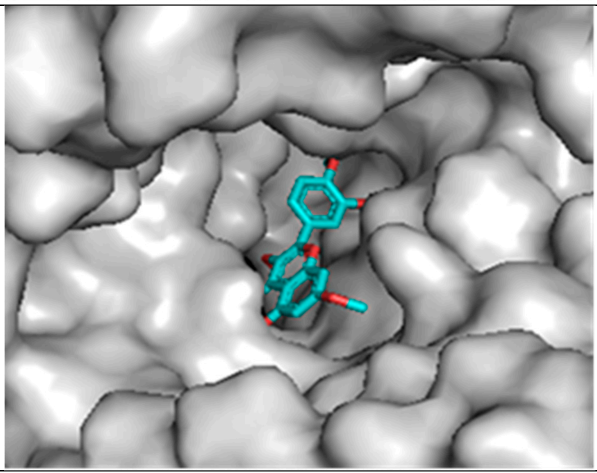

12	Azaleatin	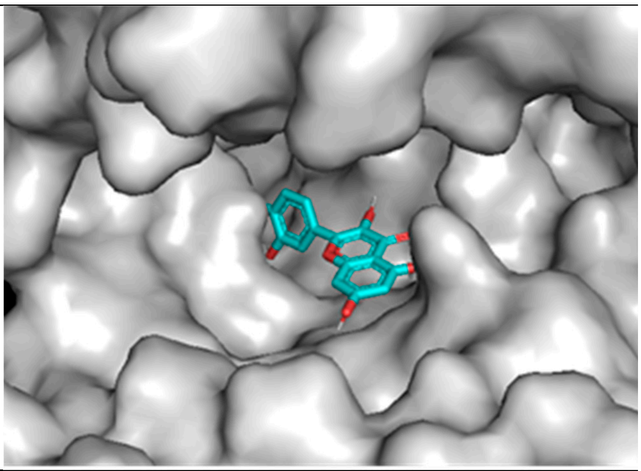
13	Ombuin	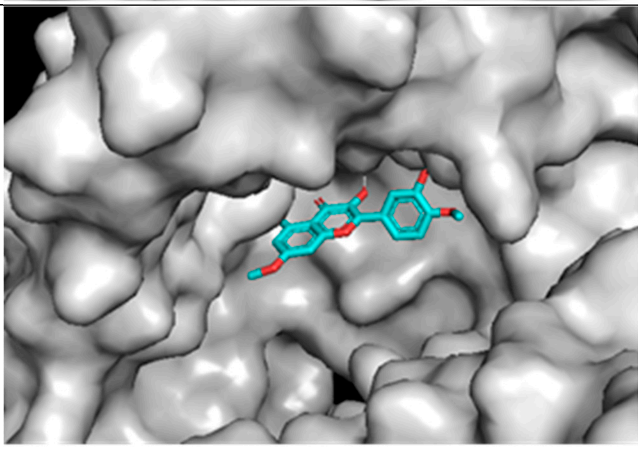
14	Pachypodol	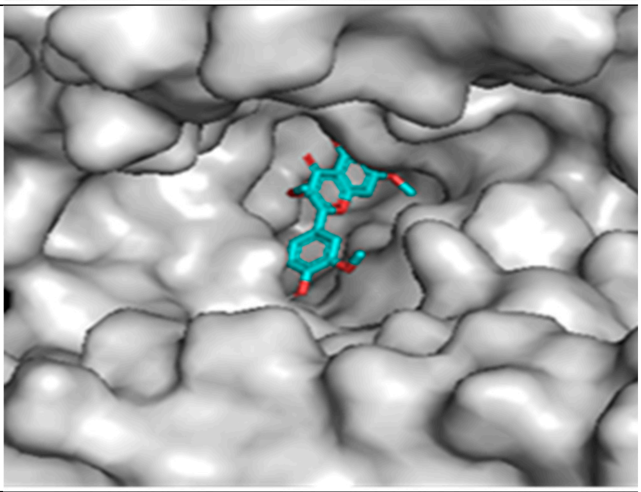
15	Retusin	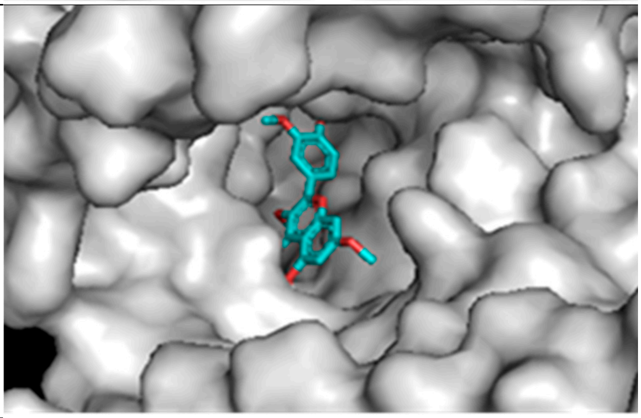

16	Rhamnazin	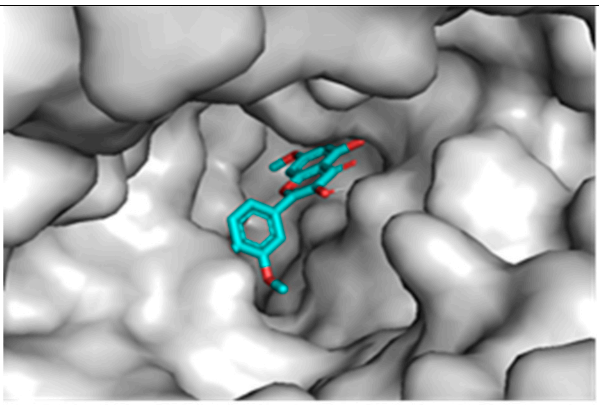
17	Eupatolitin	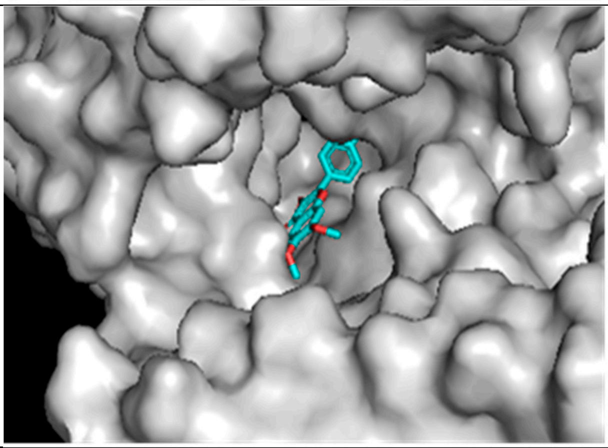

Table S1b 2D and 3D visualization of ligand binding to the protein under study.

№	Compound	Visualization ACE2 + compound in LigPlot⁺
1	Rhamnetin	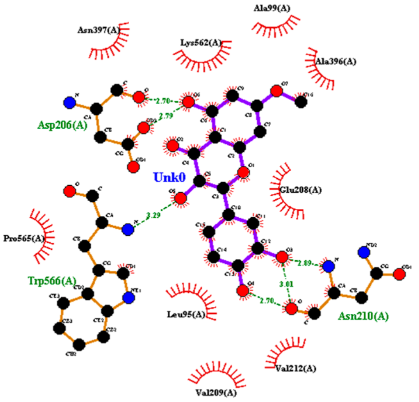 1

2	Patuletin	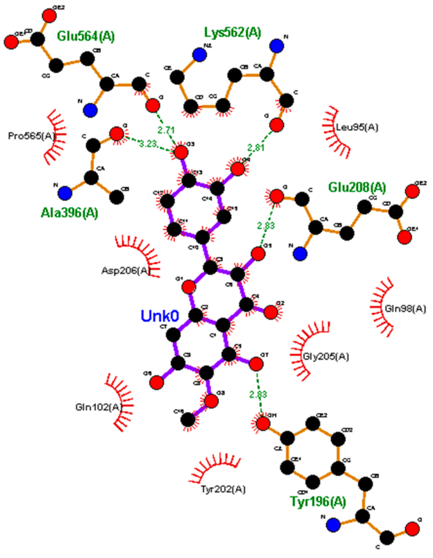<br>2
3	Isorhamnetin	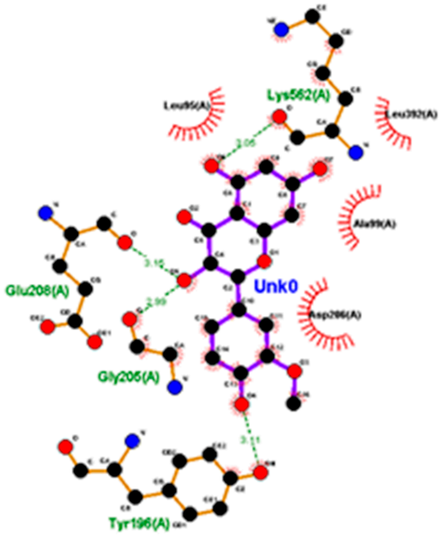<br>3
4	Tamarixetin	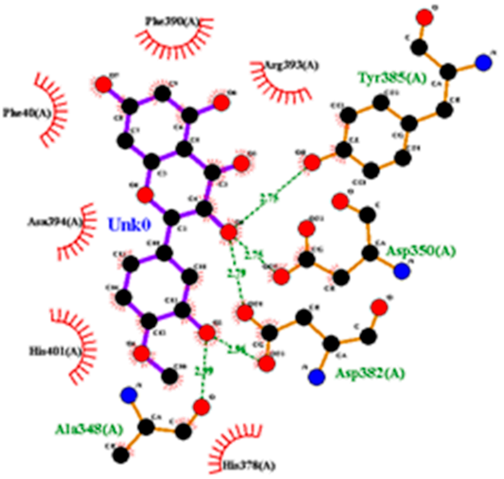<br>4

5	Quercetin	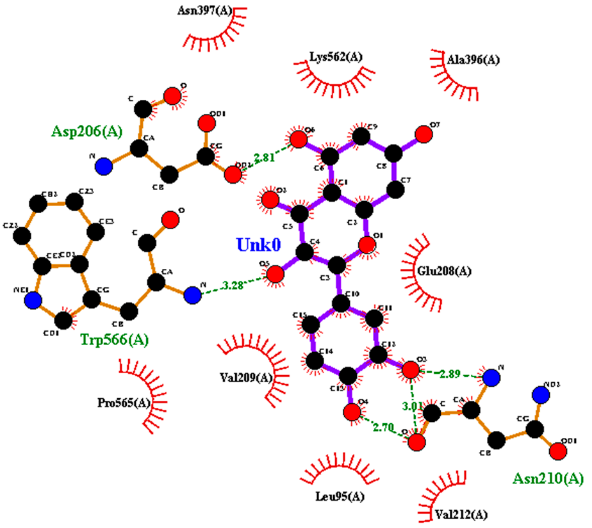<br>5
6	Corniculatus in	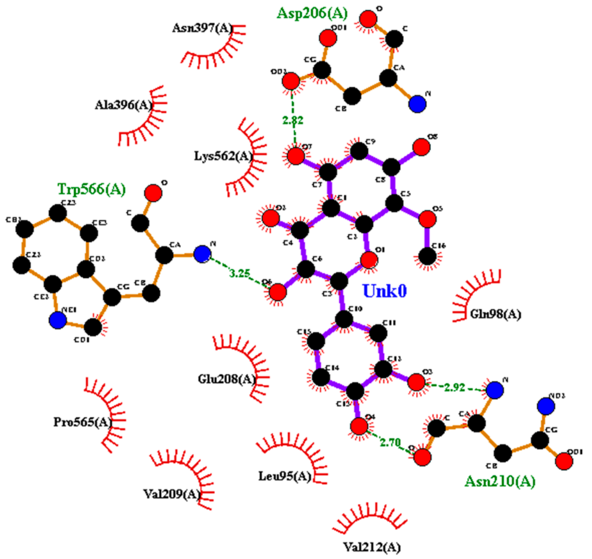<br>6

7	Dillenetin	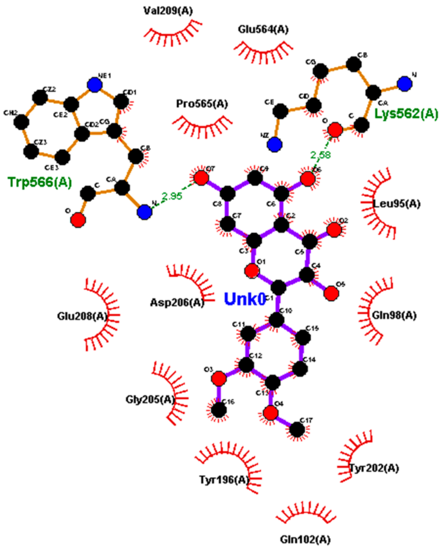<br>7
8	Nobiletin	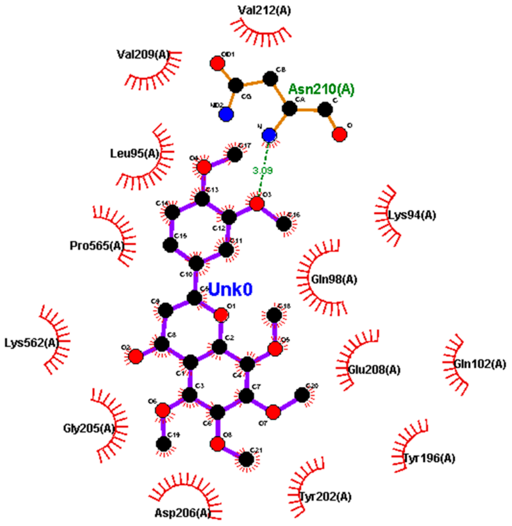<br>8

<table>
<tr><td>9</td><td>Hesperidin</td><td>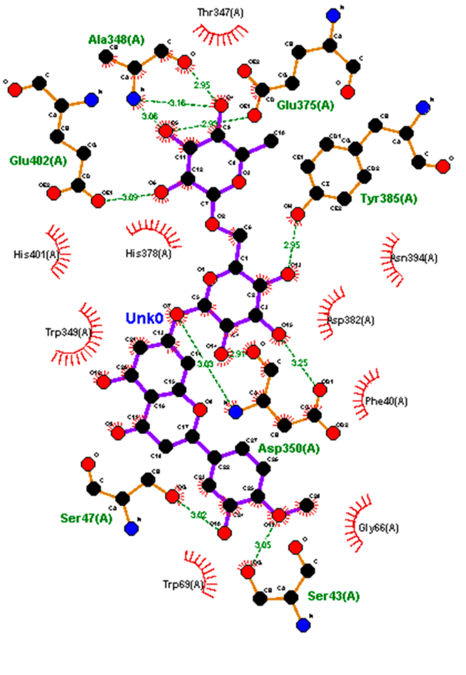

9</td></tr>
<tr><td>10</td><td>Baicalein</td><td>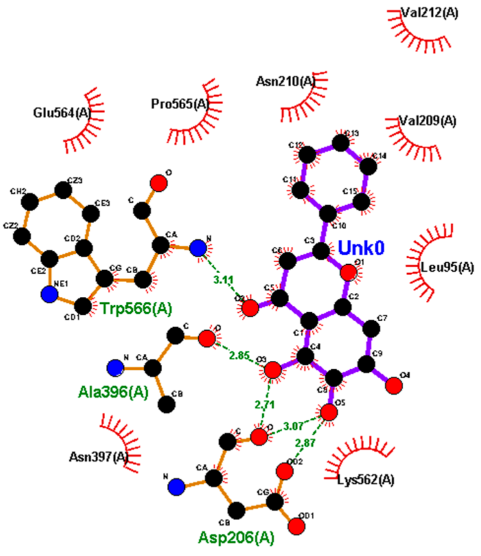

10</td></tr>
</table>

11	Ayanin	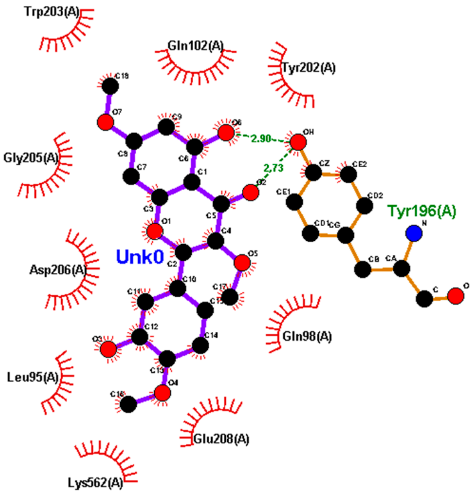<br>11
12	Azaleatin	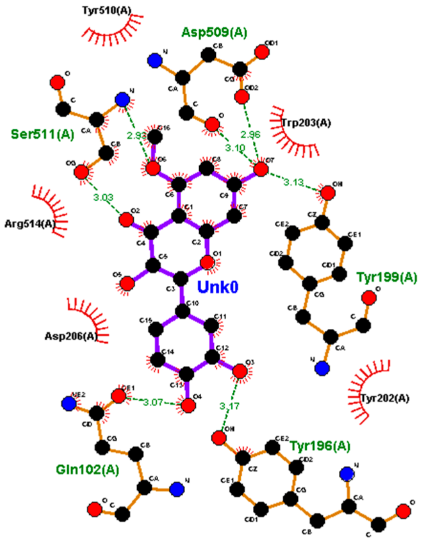<br>12
13	Ombuin	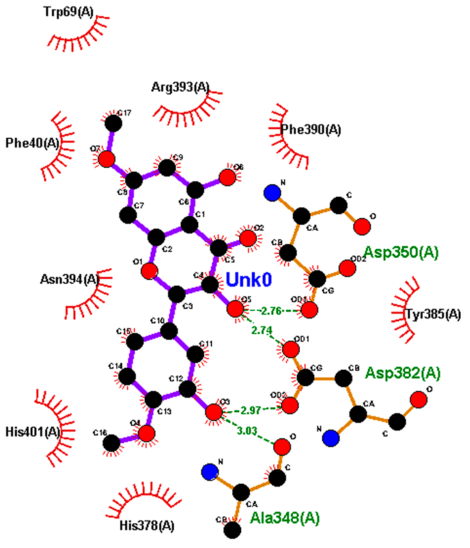<br>13

14	Pachypodol	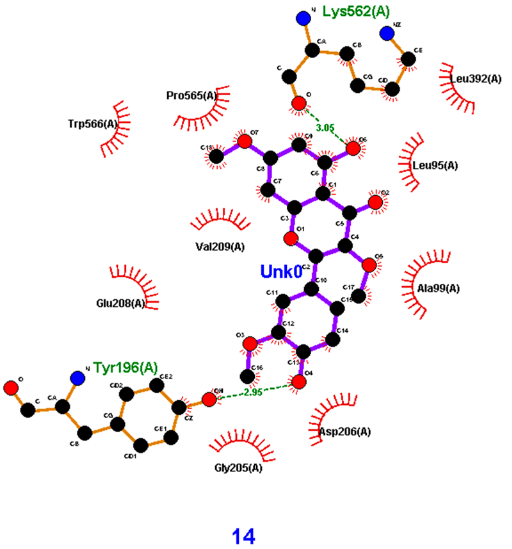<br>14
15	Retusin	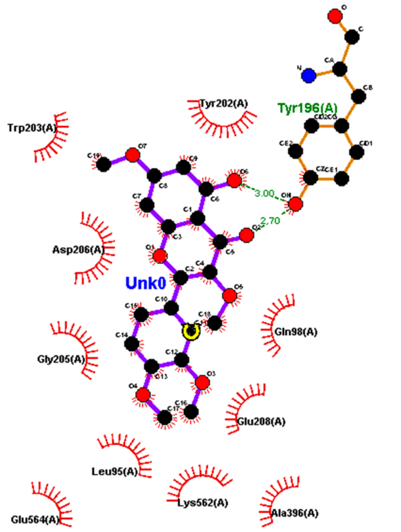<br>15
16	Rhamnazin	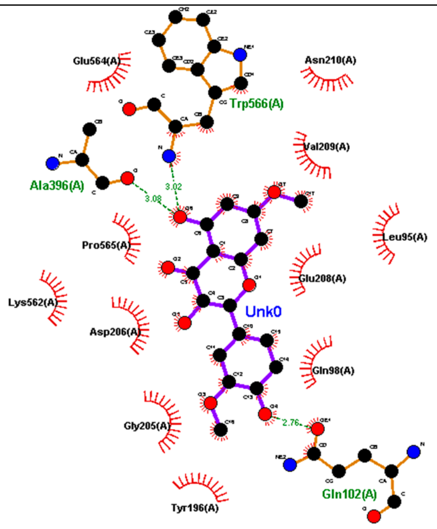<br>16

17	Eupatolitin	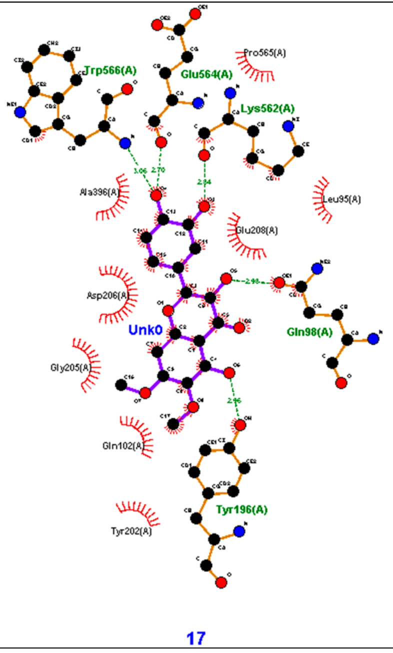

Table S1c 2D and 3D visualization of ligand binding to the protein under study.

№	Compound	Visualization ACE2 + compound in RasMol
1	Rhamnetin	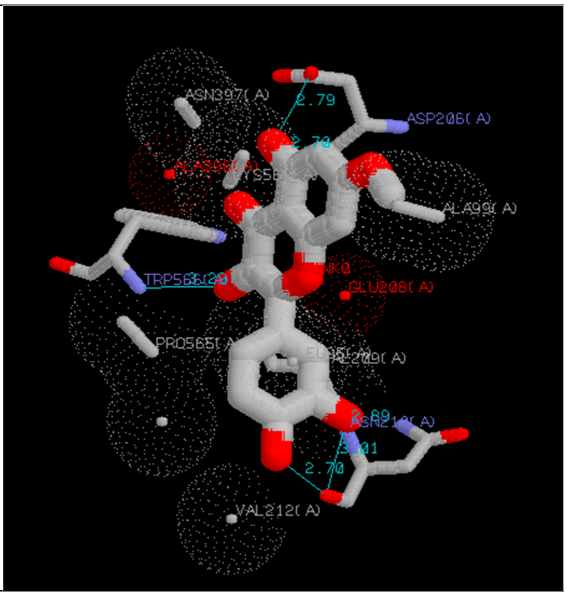

2	Patuletin	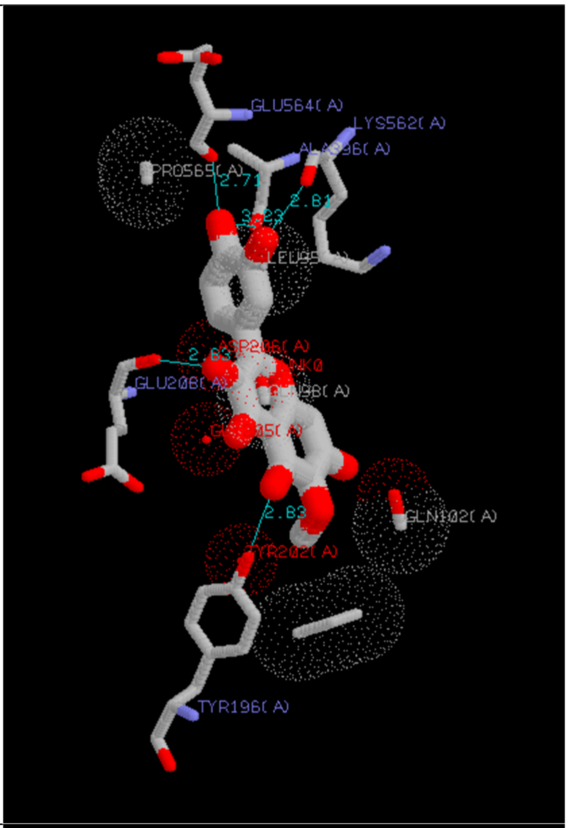
3	Isorhamnetin	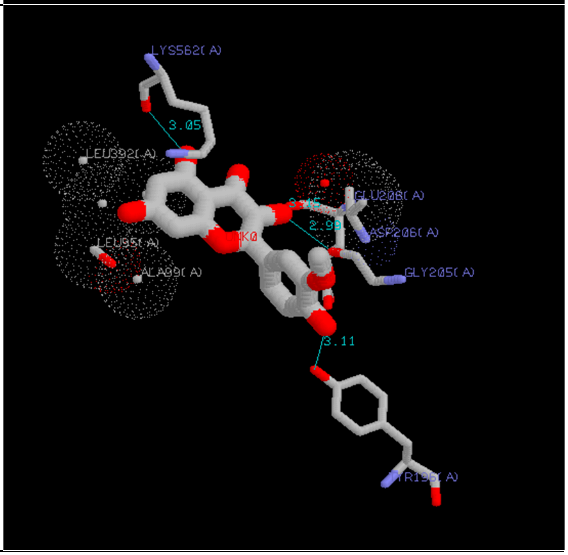

4	Tamarixetin	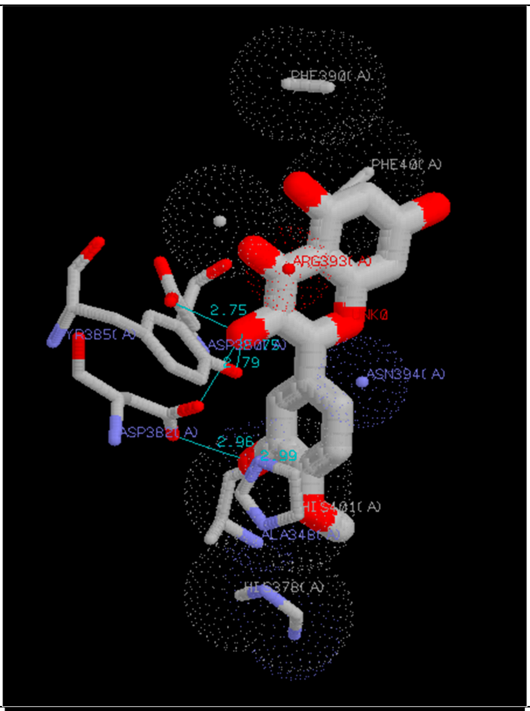
5	Quercetin	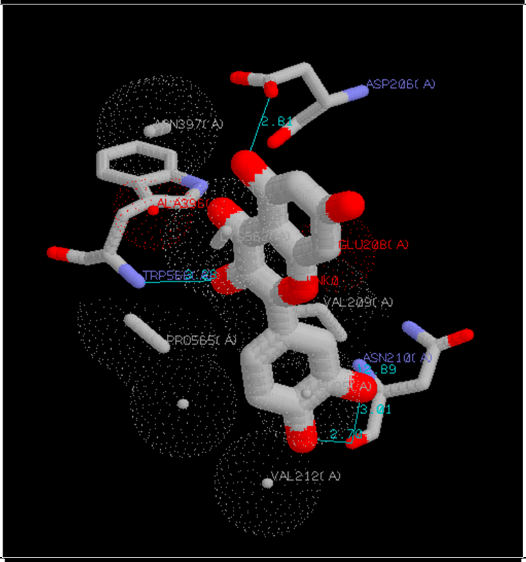
6	Corniculatusin	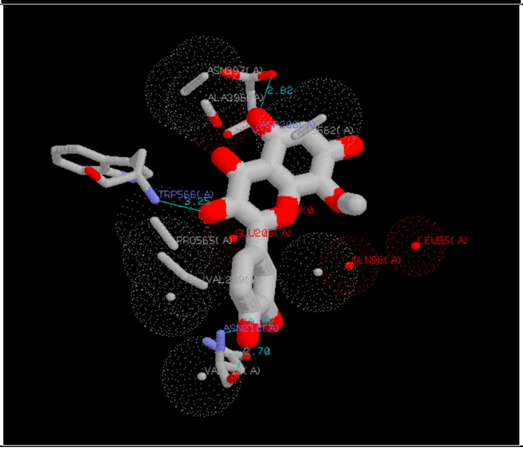

7	Dillenetin	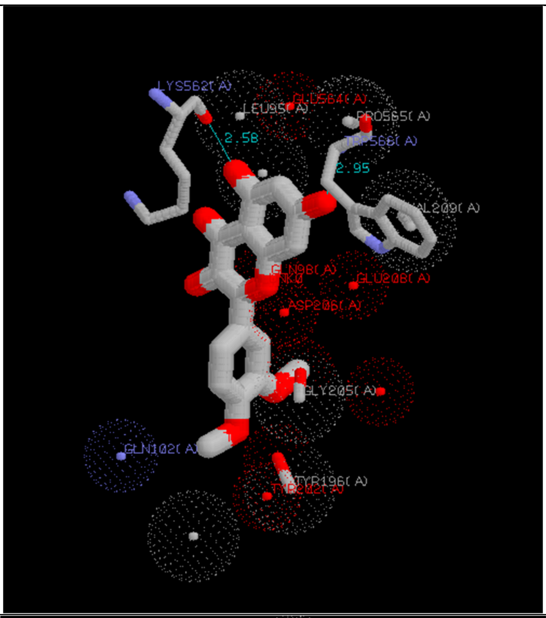
8	Nobiletin	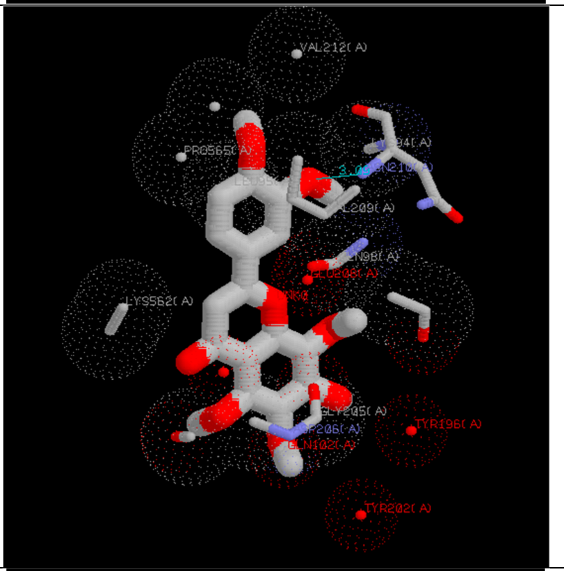
9	Hesperidin	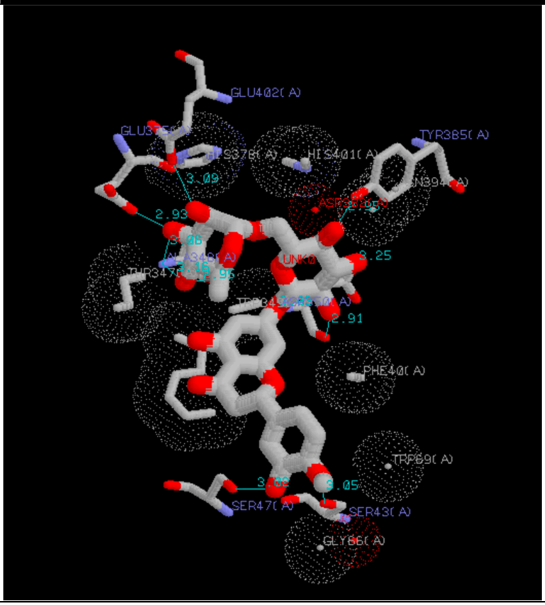

10	Baicalein	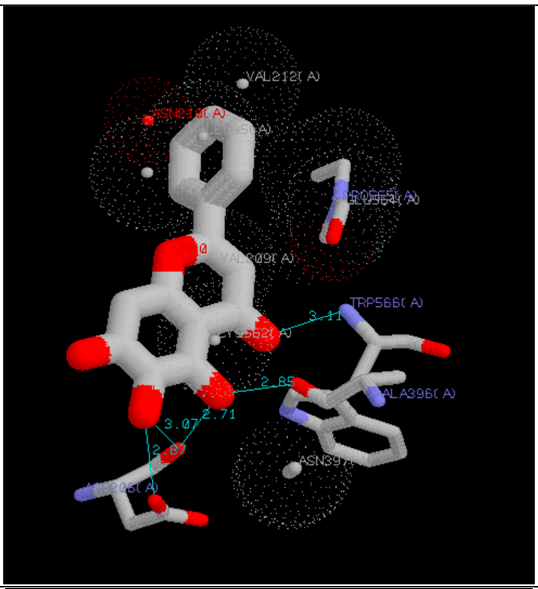
11	Ayanin	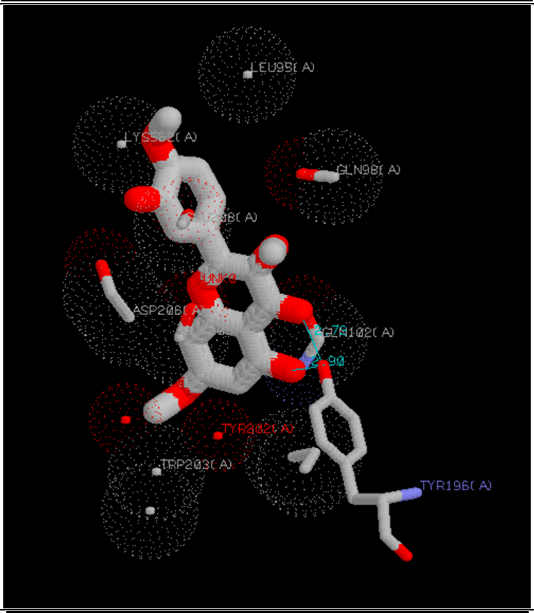
12	Azaleatin	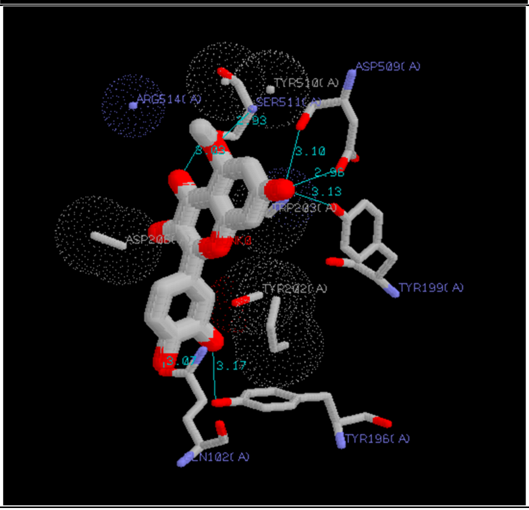

<table>
<tr><td>13</td><td>Ombuin</td><td>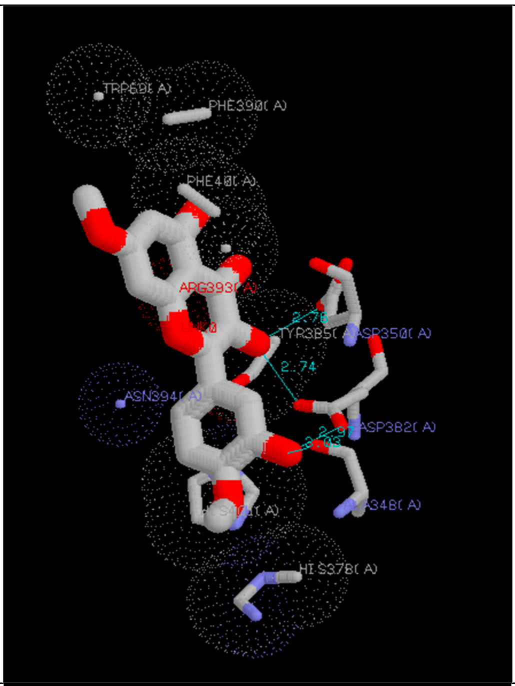
</td></tr>
<tr><td>14</td><td>Pachypodol</td><td>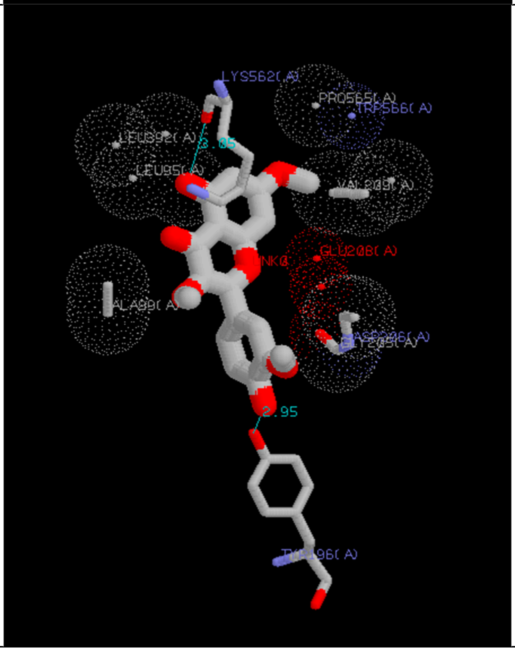
</td></tr>
</table>

15	Retusin	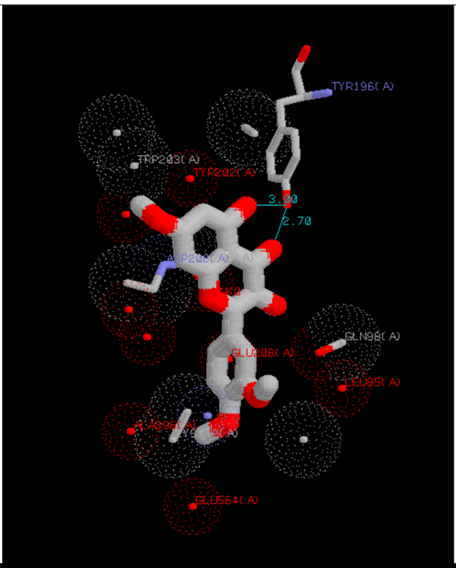
16	Rhamnazin	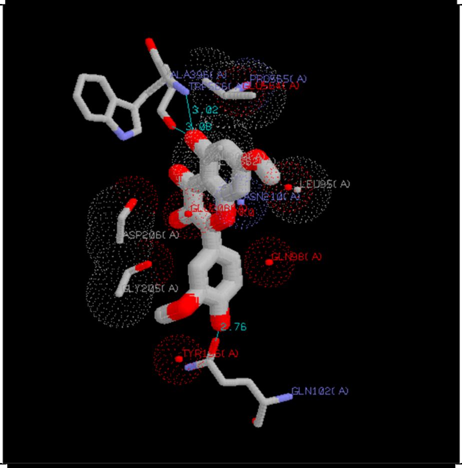
17	Eupatolitin	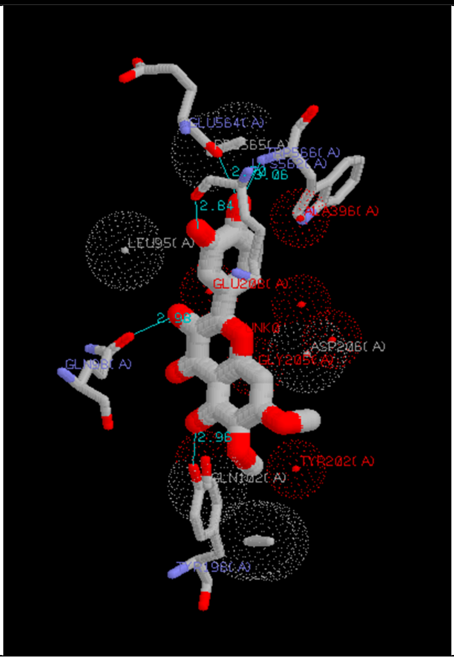

Table S2 Comparison of Toxicity Risks and Drug Score of compounds **1-17**.

**Compound**	**MW** [g/mole]	**Toxicity Risks**[a]				**Drug Score Calculations**[b]			
		MUT	TUM	IRRI	REP	cLog*P*	cLog*S*	DL	DS
**1**	316.26	red	green	green	green	1.77	-2.8	1.7	0.49
**2**	332.26	red	green	green	green	1.42	-2.51	1.5	0.49
**3**	316.26	red	green	green	green	1.77	-2.8	1.17	0.47
**4**	316.26	red	green	green	green	1.77	-2.8	1.78	0.49
**5**	302.24	red	red	green	green	1.49	-2.49	1.6	0.3
**6**	332.26	red	green	green	green	1.42	-2.51	1.5	0.49
**7**	330.29	red	green	green	green	2.04	-3.12	3.0	0.5
**8**	418.40	red	red	green	green	2.45	-3.87	2.77	0.26
**9**	610.57	green	green	green	green	-0.81	-2.75	3.46	0.57
**10**	270.24	green	green	green	green	2.34	-2.86	0.75	0.75
**11**	344.32	red	green	green	green	2.47	-3.25	1.65	0.46
**12**	316.26	red	green	green	green	2.04	-3.12	2.0	0.48
**13**	330.29	red	green	green	green	2.04	-3.12	1.78	0.48
**14**	344.32	red	green	green	green	2.47	-3.25	1.03	0.43
**15**	358.35	red	green	green	green	2.75	-3.56	2.88	0.46
**16**	330.29	red	green	green	green	2.04	-3.12	1.17	0.45
**17**	346.29	red	green	green	green	1.7	-2.82	1.5	0.47

[a] Not toxic (green), highly toxic: (red), slightly toxic: (yellow). REP: Reproductive effective, IRRIT: Irritant, TUM: Tumorigenic, MUT: Mutagenic. [b] DS: Drug-Score, DL: Drug Likeness, Sol: Solubility.

Table S3 Molinspiration calculations for compounds **1-17**.

**Co**	**3D Molecular Structure**	**Molecular properties**		**Drug scores**	
**1**	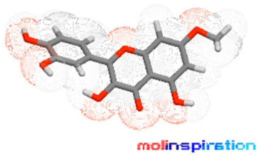	miLogP	2.22	GPCR ligand	-0.11
		TPSA	120.36	Ion channel modulator	-0.27
		MW	316.26	Kinase inhibitor	0.21
		nOHNH	4	Nuclear receptor ligand	0.27
		nviolations	0	Protease inhibitor	-0.27
		volume	257.61	Enzyme inhibitor	0.20

**2**	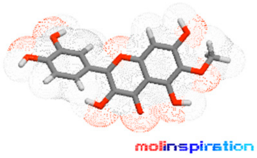	miLogP 1.70<br>TPSA 140.59<br>MW 332.26<br>nOHNH 5<br>nviolations 0<br>nrotb 2<br>volume 265.63	GPCR ligand -0.14<br>Ion channel modulator -0.34<br>Kinase inhibitor 0.21<br>Nuclear receptor ligand 0.13<br>Protease inhibitor -0.35<br>Enzyme inhibitor 0.17
**3**	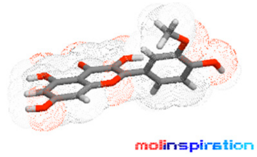	miLogP 1.99<br>TPSA 120.36<br>MW 316.26<br>nOHNH 4<br>nviolations 0<br>volume 257.61	GPCR ligand -0.10<br>Ion channel modulator -0.26<br>Kinase inhibitor 0.25<br>Nuclear receptor ligand 0.28<br>Protease inhibitor -0.30<br>Enzyme inhibitor 0.22
**4**	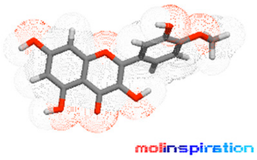	miLogP 1.99<br>TPSA 120.36<br>MW 316.26<br>nOHNH 4<br>nviolations 0<br>volume 257.61	GPCR ligand -0.10<br>Ion channel modulator -0.26<br>Kinase inhibitor 0.25<br>Nuclear receptor ligand 0.28<br>Protease inhibitor -0.30<br>Enzyme inhibitor 0.22
**5**	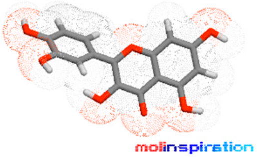	miLogP 1.68<br>TPSA 131.35<br>MW 302.24<br>nOHNH 5<br>nviolations 0<br>volume 240.08	GPCR ligand -0.06<br>Ion channel modulator -0.19<br>Kinase inhibitor 0.28<br>Nuclear receptor ligand 0.36<br>Protease inhibitor -0.25<br>Enzyme inhibitor 0.28
**6**	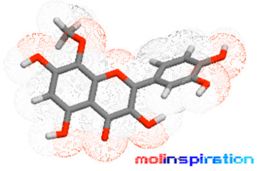	miLogP 1.70<br>TPSA 140.59<br>MW 332.26<br>nOHNH 5<br>nviolations 0<br>volume 265.63	GPCR ligand -0.15<br>Ion channel modulator -0.12<br>Kinase inhibitor 0.17<br>Nuclear receptor ligand 0.15<br>Protease inhibitor -0.29<br>Enzyme inhibitor 0.22
**7**	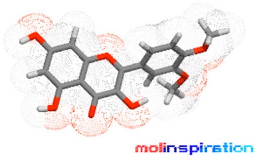	miLogP 2.30<br>TPSA 109.36<br>MW 330.29<br>nOHNH 3<br>nviolations 0<br>volume 275.14	GPCR ligand -0.10<br>Ion channel modulator -0.26<br>Kinase inhibitor 0.23<br>Nuclear receptor ligand 0.27<br>Protease inhibitor -0.26<br>Enzyme inhibitor 0.19
**8**	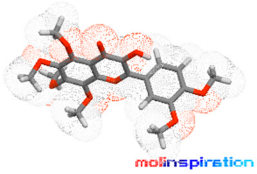	miLogP 3.08<br>TPSA 105.84<br>MW 418.40<br>nOHNH 1	GPCR ligand -0.15<br>Ion channel modulator -0.10<br>Kinase inhibitor 0.11<br>Nuclear receptor ligand 0.03<br>Protease inhibitor -0.26

		nviolations 0<br>volume 361.29	Enzyme inhibitor 0.15
**9**	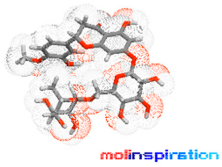	miLogP -0.55<br>TPSA 234.30<br>MW 610.57<br>nOHNH 8<br>nviolations 3<br>volume 511.79	GPCR ligand -0.01<br>Ion channel modulator -0.59<br>Kinase inhibitor -0.36<br>Nuclear receptor ligand -0.20<br>Protease inhibitor 0.00<br>Enzyme inhibitor 0.06
**10**	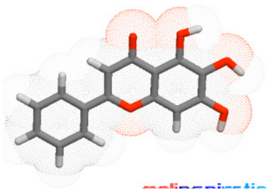	miLogP 2.68<br>TPSA 90.89<br>MW 270.24<br>nOHNH 3<br>nviolations 0<br>volume 224.05	GPCR ligand -0.12<br>Ion channel modulator -0.18<br>Kinase inhibitor 0.19<br>Nuclear receptor ligand 0.17<br>Protease inhibitor -0.35<br>Enzyme inhibitor 0.26
**11**	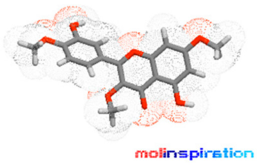	miLogP 2.80<br>TPSA 98.37<br>MW 344.32<br>nOHNH 2<br>nviolations 0<br>volume 292.67	GPCR ligand -0.12<br>Ion channel modulator -0.22<br>Kinase inhibitor 0.14<br>Nuclear receptor ligand 0.14<br>Protease inhibitor -0.27<br>Enzyme inhibitor 0.16
**12**	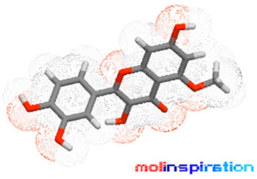	miLogP 1.96<br>TPSA 120.36<br>MW 316.26<br>nOHNH 4<br>nviolations 0<br>volume 275.61	GPCR ligand -0.07<br>Ion channel modulator -0.24<br>Kinase inhibitor 0.24<br>Nuclear receptor ligand 0.29<br>Protease inhibitor -0.28<br>Enzyme inhibitor 0.19
**13**	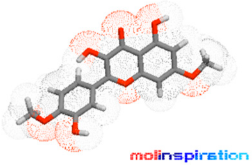	miLogP 2.53<br>TPSA 109.36<br>MW 330.29<br>nOHNH 3<br>nviolations 0<br>volume 275.14	GPCR ligand -0.12<br>Ion channel modulator -0.28<br>Kinase inhibitor 0.21<br>Nuclear receptor ligand 0.23<br>Protease inhibitor -0.27<br>Enzyme inhibitor 0.18
**14**	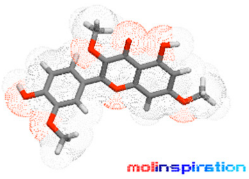	miLogP 2.80<br>TPSA 98.37<br>MW 344.32<br>nOHNH 2<br>nviolations 0<br>volume 292.67	GPCR ligand -0.12<br>Ion channel modulator -0.22<br>Kinase inhibitor 0.14<br>Nuclear receptor ligand 0.14<br>Protease inhibitor -0.27<br>Enzyme inhibitor 0.16

**15**	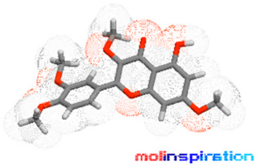	miLogP 3.11<br>TPSA 87.38<br>MW 358.35<br>nOHNH 1<br>nviolations 0<br>volume 310.20	GPCR ligand -0.13<br>Ion channel modulator -0.22<br>Kinase inhibitor 0.12<br>Nuclear receptor ligand 0.13<br>Protease inhibitor -0.25<br>Enzyme inhibitor 0.14
**16**	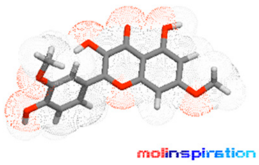	miLogP 2.53<br>TPSA 109.36<br>MW 330.29<br>nOHNH 3<br>nviolations 0<br>volume 275.14	GPCR ligand -0.12<br>Ion channel modulator -0.28<br>Kinase inhibitor 0.21<br>Nuclear receptor ligand 0.23<br>Protease inhibitor -0.27<br>Enzyme inhibitor 0.18
**17**	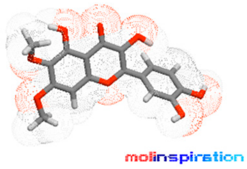	miLogP 2.01<br>TPSA 129.59<br>MW 346.29<br>nOHNH 4<br>nviolations 0<br>volume 283.16	GPCR ligand -0.15<br>Ion channel modulator -0.34<br>Kinase inhibitor 0.21<br>Nuclear receptor ligand 0.09<br>Protease inhibitor -0.35<br>Enzyme inhibitor 0.15

Molecular weight in g/mol
Topological polar surface area in Å²
Volume in Å3

Table S4 Comparison of Molinspiration data for compounds **1-17**.

Compd	Lipinski parameters calculations[a]				Drug-likeness[b]					
	TPSA	NONH	NV	VOL	GPCRL	ICM	KI	NRL	PI	EI
**1**	120.36	4	0	257.61	-0.11	-0.27	0.21	0.27	-0.27	0.20
**2**	140.59	5	0	265.63	-0.14	-0.34	0.21	0.13	-0.35	0.17
**3**	120.36	4	0	257.61	-0.10	-0.26	0.25	0.28	-0.30	0.22
**4**	120.36	4	0	257.61	-0.10	-0.26	0.25	0.28	-0.30	0.22
**5**	131.35	5	0	240.08	-0.06	-0.19	0.28	0.36	-0.25	0.28
**6**	140.59	5	0	265.63	-0.15	-0.12	0.17	0.15	-0.29	0.22
**7**	109.36	3	0	275.14	0.10	-0.26	0.23	0.27	-0.26	0.19
**8**	105.84	1	0	361.29	-0.15	-0.10	0.11	0.03	-0.26	0.15
**9**	234.30	8	3	511.79	-0.01	-0.59	0.36	-0.20	0.00	0.06
**10**	90.89	3	0	224.05	-0.12	-0.18	0.19	0.17	-0.35	0.26

**11**	98.37	2	0	292.67	-0.12	-0.22	0.14	0.14	-0.27	0.16
**12**	120.36	4	0	275.61	-0.07	-0.24	0.24	0.29	-0.28	0.19
**13**	109.36	3	0	275.14	-0.12	-0.28	0.21	0.23	-0.27	0.18
**14**	98.37	2	0	292.67	-0.12	-0.22	0.14	0.14	-0.27	0.16
**15**	87.38	1	0	310.20	-0.13	-0.22	0.12	0.13	-0.25	0.14
**16**	109.36	3	0	275.14	-0.12	-0.28	0.21	0.23	-0.27	0.18
**17**	129.59	4	0	283.16	-0.15	-0.34	0.21	0.09	-0.35	0.15

Molecular weight in g/mol
Topological polar surface area in Å²

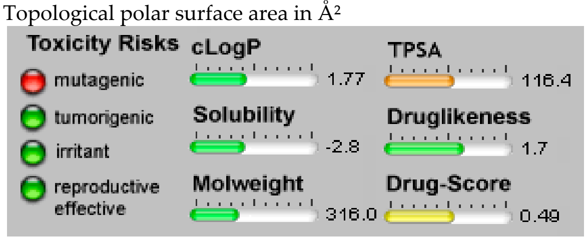

Compound **1**

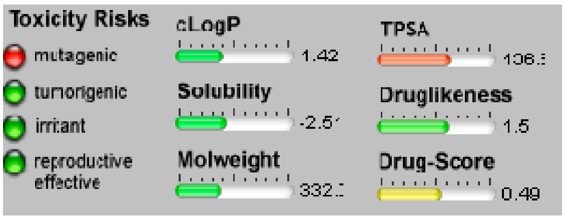

Compound **2**

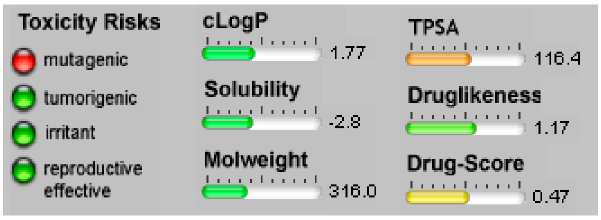

Compound **3**

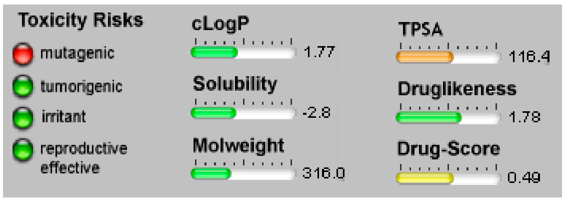

Compound **4**

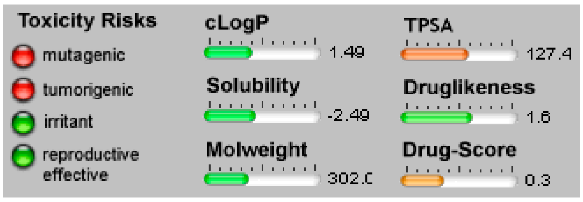

Compound **5**

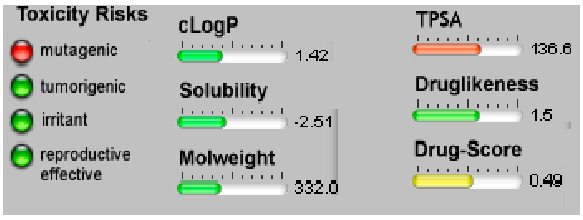

Compound **6**

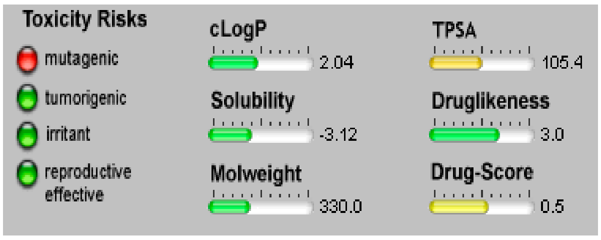

Compound **7**

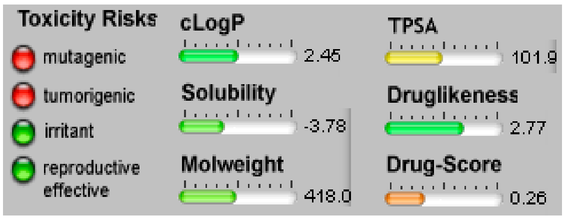

Compound **8**

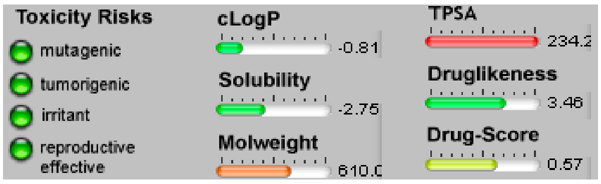

Compound **9**

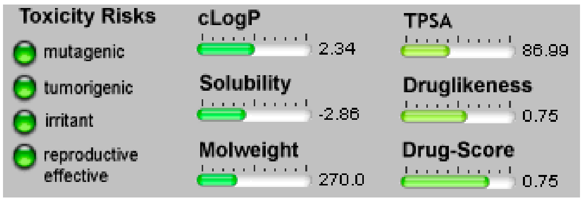

Compound **10**

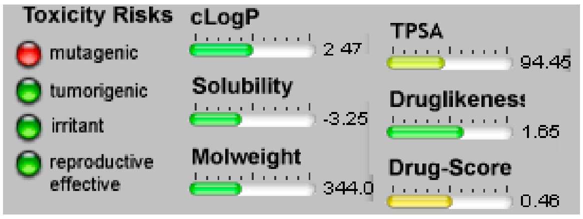

Compound **11**

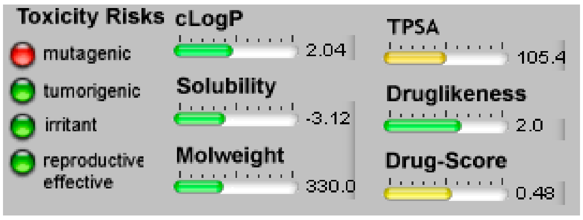

Compound **12**

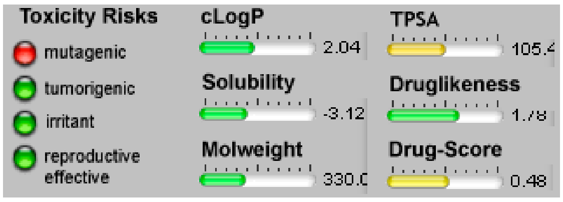

Compound **13**

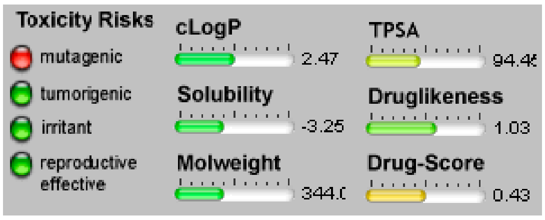

Compound **14**

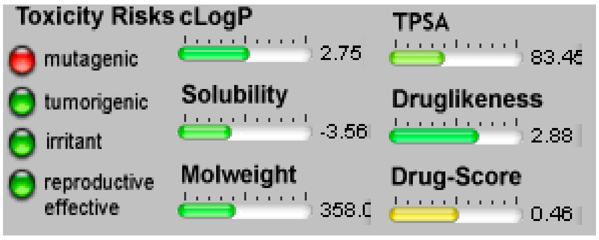

Compound **15**

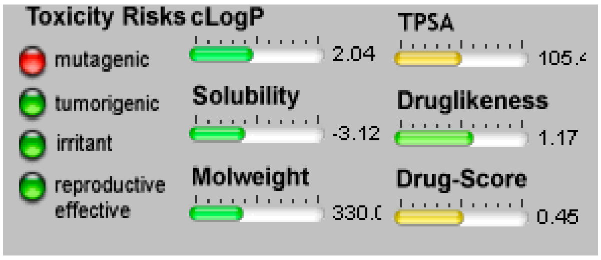

Compound **16**

Not toxic (), highly toxic: (),

slightly toxic: (). REP: Reproductive effective, IRRIT: Irritant, TUM: Tumorigenic, MUT: Mutagenic.

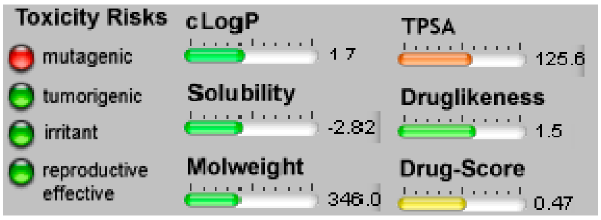

Compound **17**

Drug-Scores:

DS: Drug-Score, DL: Drug Likeness, Sol: Solubility.

Toxicity Risks

Figure S1. Osiris calculations of likeness and drug-score of compounds (**1-17**).

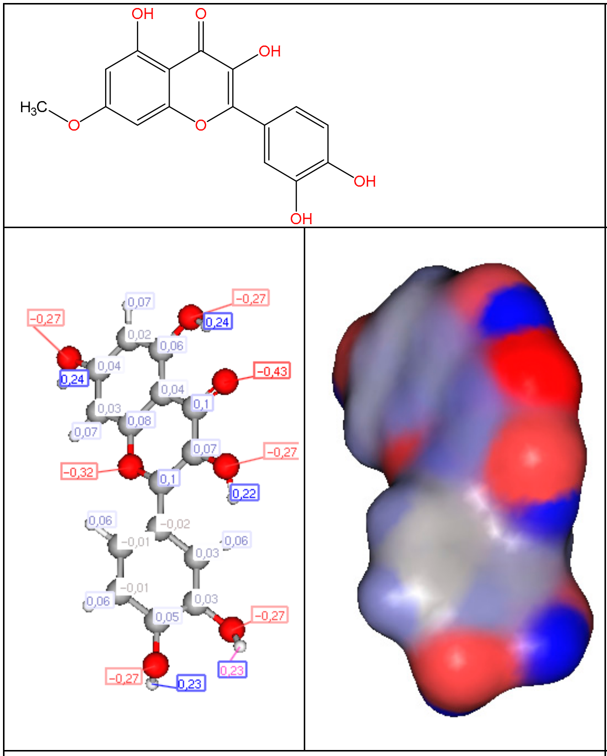

Compound **1**

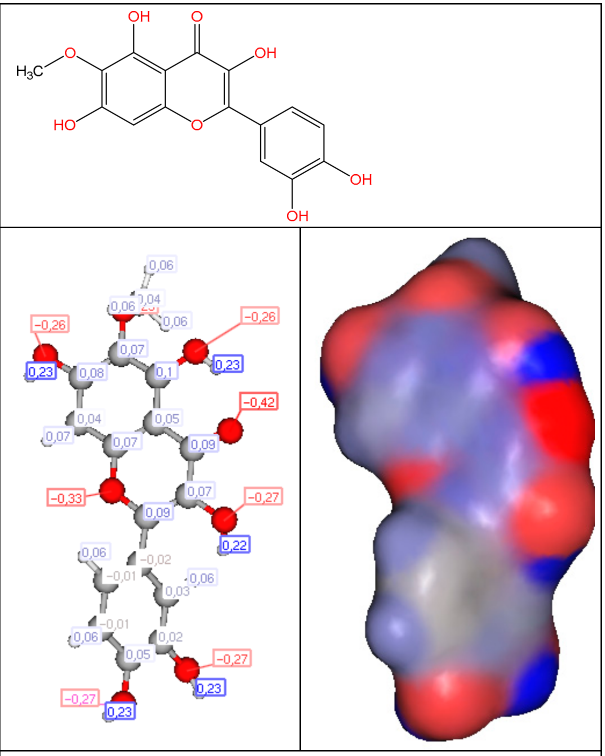

Compound **2**

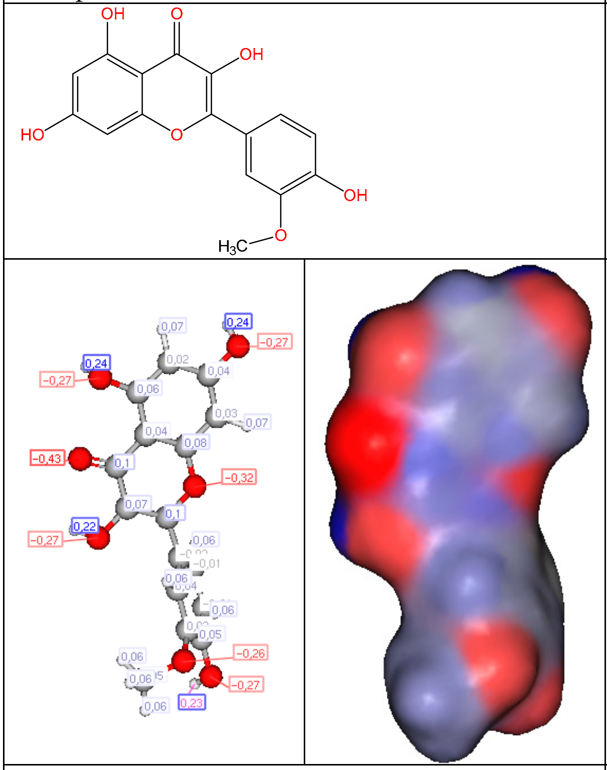

Compound **3**

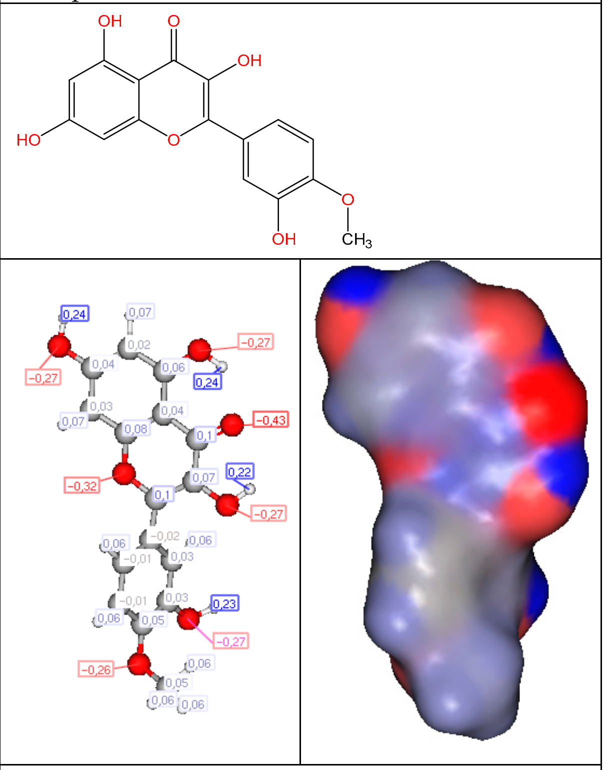

Compound **4**

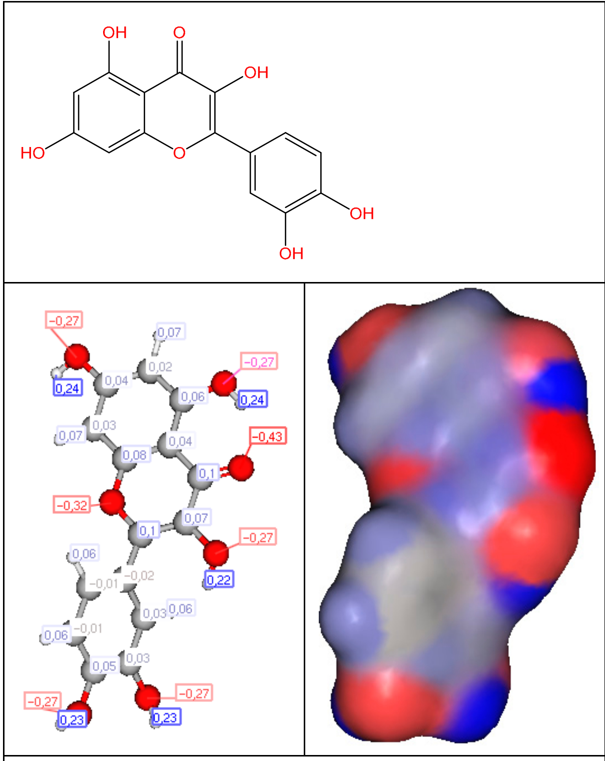

Compound **5**

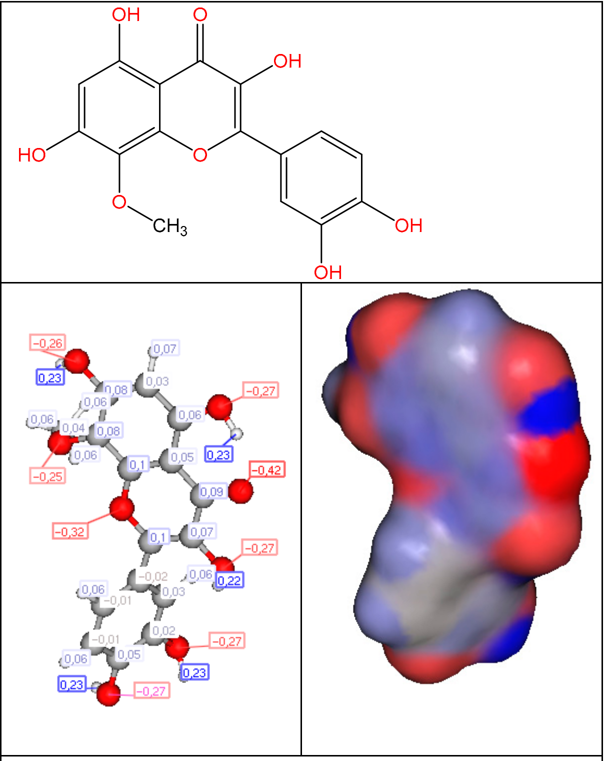

Compound **6**

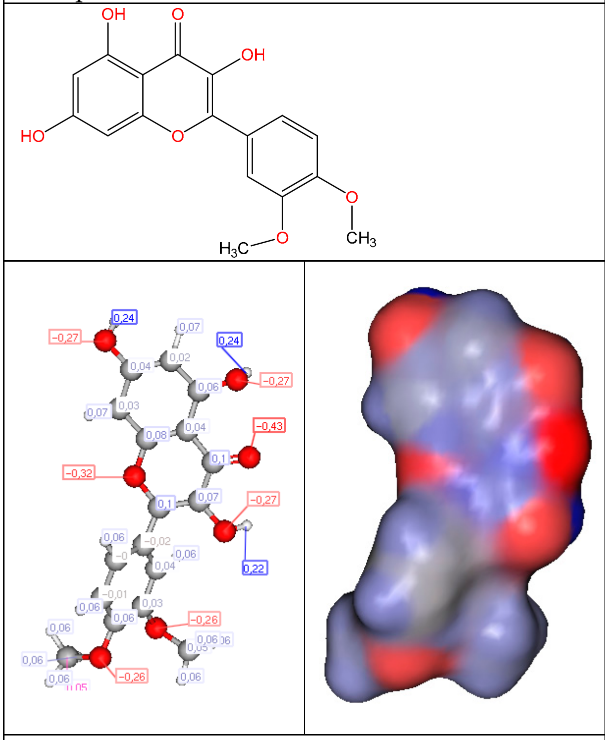

Compound **7**

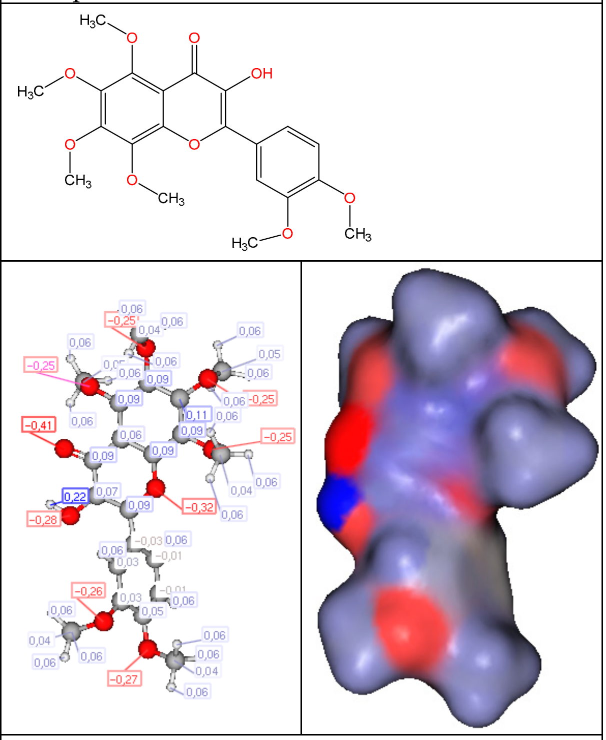

Compound **8**

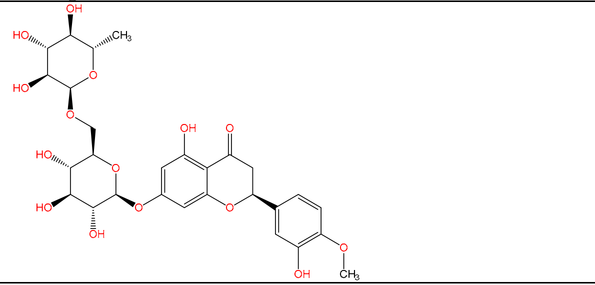

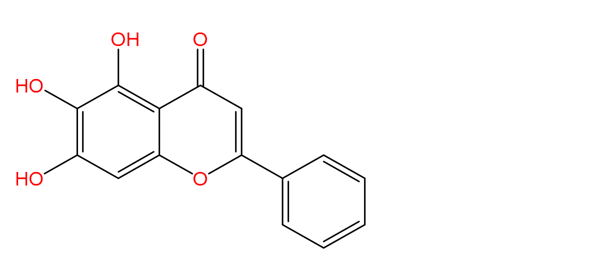

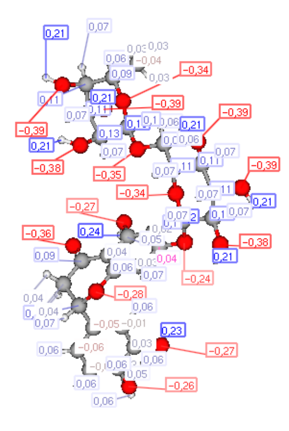 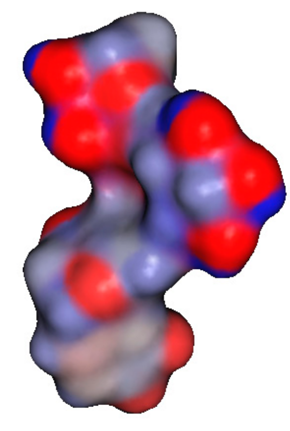	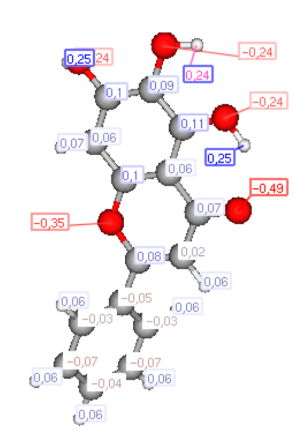 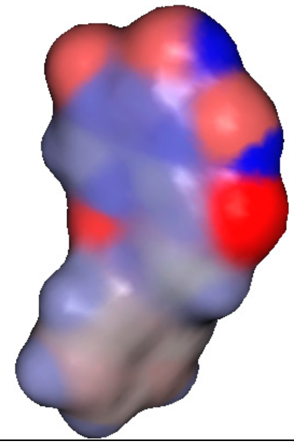
Compound **9**	Compound **10**
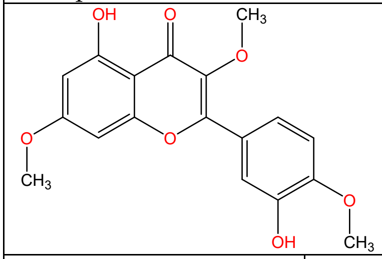	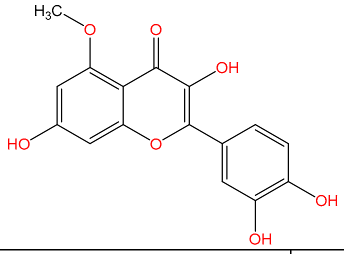
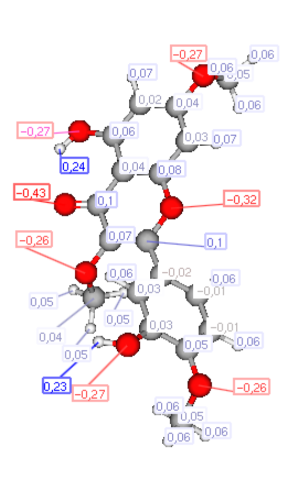 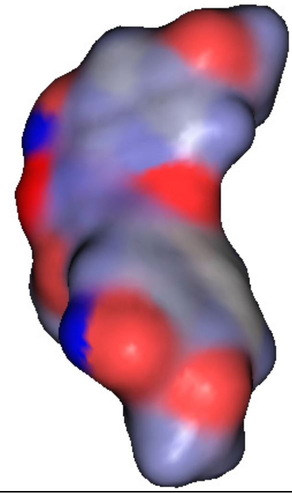	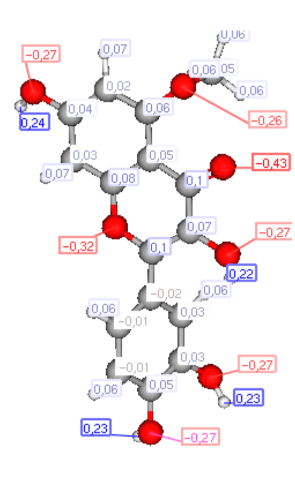 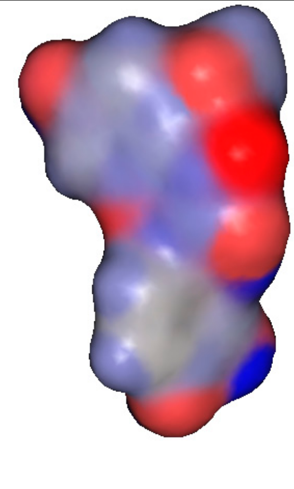
Compound **11**	Compound **12**
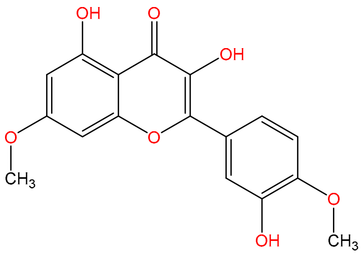	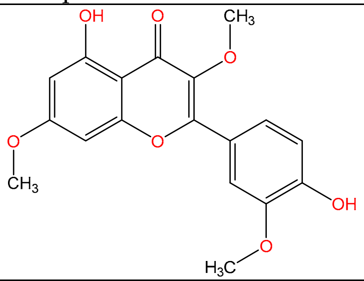

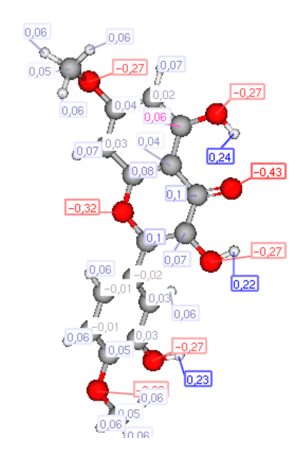	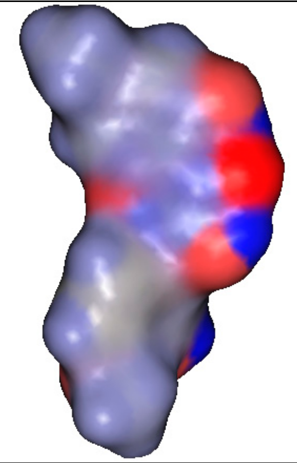	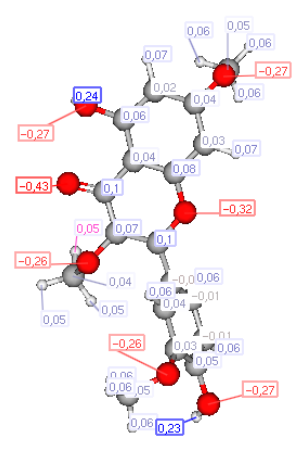	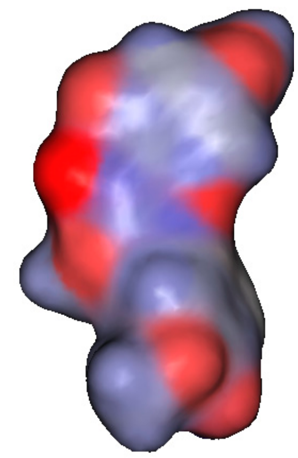
Compound **13**		Compound **14**	
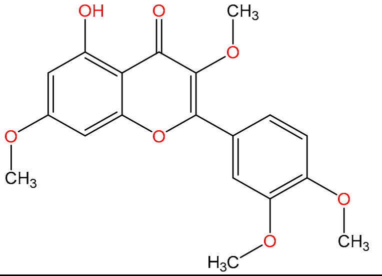		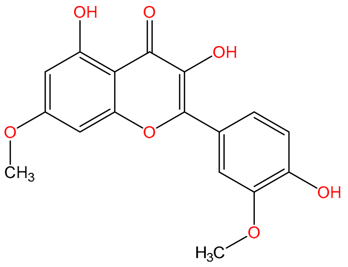	
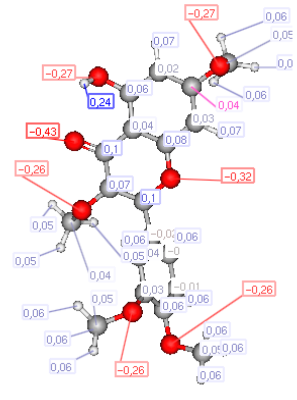	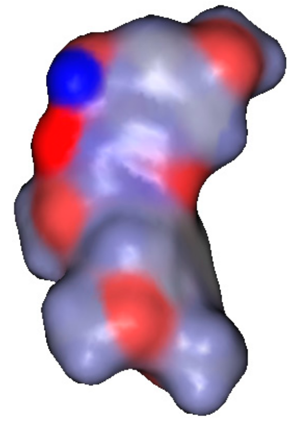	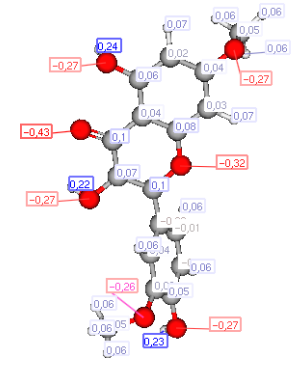	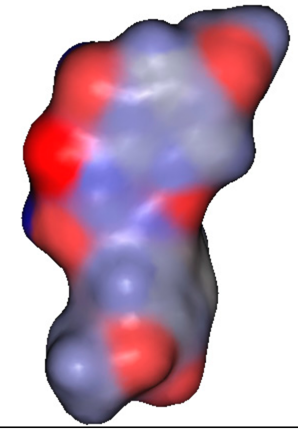
Compound **15**		Compound **16**	
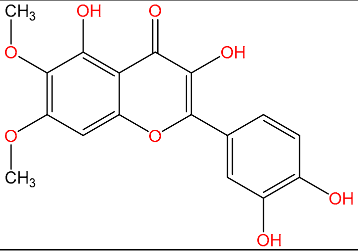			

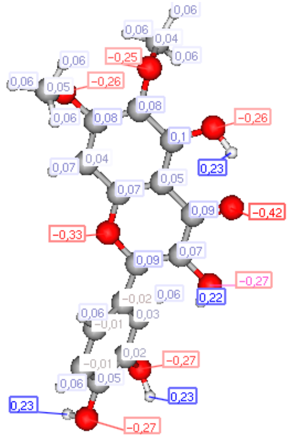	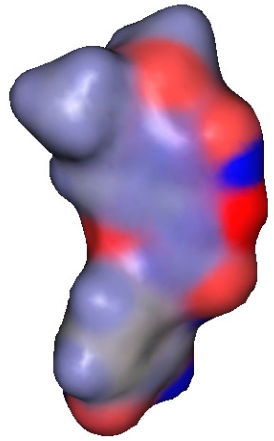	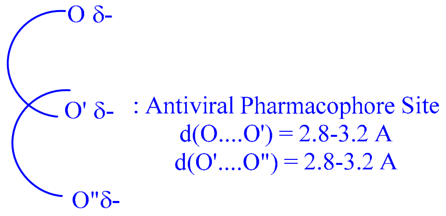
Compound **17**		**Topology of antiviral pharmacophore site of 1-17**

Figure S2. Atomic charge calculations for compounds **1-17**.
